# Molecular crosstalk between MUC1 and STAT3 influences the anti-proliferative effect of Napabucasin in epithelial cancers

**DOI:** 10.1038/s41598-024-53549-4

**Published:** 2024-02-07

**Authors:** Mukulika Bose, Alexa Sanders, Aashna Handa, Aabha Vora, Manuel R. Cardona, Cory Brouwer, Pinku Mukherjee

**Affiliations:** 1 Department of Biological Sciences, UNC Charlotte, Charlotte, NC 28223 USA; 2 Department of Bioinformatics, UNC Charlotte, Charlotte, NC 28223 USA

**Keywords:** MUC1, STAT3, Napabucasin, Gastrointestinal cancers, Precision medicine, Cancer, Cancer therapy, Gastrointestinal cancer, Oncogenes, Tumour biomarkers

## Abstract

MUC1 is a transmembrane glycoprotein that is overexpressed and aberrantly glycosylated in epithelial cancers. The cytoplasmic tail of MUC1 (MUC1 CT) aids in tumorigenesis by upregulating the expression of multiple oncogenes. Signal transducer and activator of transcription 3 (STAT3) plays a crucial role in several cellular processes and is aberrantly activated in many cancers. In this study, we focus on recent evidence suggesting that STAT3 and MUC1 regulate each other’s expression in cancer cells in an auto-inductive loop and found that their interaction plays a prominent role in mediating epithelial-to-mesenchymal transition (EMT) and drug resistance. The STAT3 inhibitor Napabucasin was in clinical trials but was discontinued due to futility. We found that higher expression of MUC1 increased the sensitivity of cancer cells to Napabucasin. Therefore, high-MUC1 tumors may have a better outcome to Napabucasin therapy. We report how MUC1 regulates STAT3 activity and provide a new perspective on repurposing the STAT3-inhibitor Napabucasin to improve clinical outcome of epithelial cancer treatment.

## Introduction

STAT3 belongs to a family of transcription factors comprising STAT1, STAT2, STAT3, STAT4, STAT5A, STAT5B, and STAT6. In humans, this is encoded by the STAT3 gene. STAT3 is activated by several cytokines and growth factors such as IL-6, IL-9, IL-10, IL-27, TNF-α, MCP-1, EGF, PDGF, G-CSF, and GM-CSF. Active STAT3 plays an important role in tumorigenesis by regulating transcription of genes associated with cell development, differentiation, proliferation, survival, and angiogenesis^1^. STAT3 activation is detected in several malignancies, and its inhibition led to reversal of the malignant phenotype.

STAT3 activation has been described in nearly 70% of solid and hematological tumors^1^. STAT3 is constitutively activated by phosphorylation of Tyr705, in primary human pancreatic ductal adenocarcinomas (PDA), in PDA cell lines, and in PDA xenografts^2^. Studies in STAT3 conditional knockout mice demonstrate that the STAT3 pathway is inactive in normal pancreas, and it is not required for pancreatic development and homeostasis^2^. However, STAT3 was found to be necessary for the development of the acinar-to-ductal metaplasia process, an early event in PDA pathogenesis, which is mediated by ectopic expression of the Pdx1 transcription factor, a key regulator in early pancreatic cancer development^3^. Functional inactivation of STAT3 in a subset of PDA cell lines led to significant inhibition in cell proliferation in vitro and reduced tumor growth in vivo. Inhibition of activated STAT3 resulted in the delay of G1/S-phase progression due to inhibition of cyclin-dependent kinase 2 activity because of increased expression of p21/WAF1. Overall, the study clearly showed that with malignant transformation, activated STAT3 promotes proliferation of cells by modulating G1/S-phase progression and supports the malignant phenotype of human PDA cells^4^.

Interestingly, activation of STAT3 also suppresses tumor growth^5^ and induces differentiation and apoptosis in some contexts^6,7^. The mechanisms underlying STAT3's diverse and sometimes opposing roles are still largely unknown. It is assumed that STAT3 recruits specific co-activators and activates distinct gene expression programs based on the genetic background, type, and developmental stage of the cell^8^. This hypothesis raises an interesting issue of whether STAT3 pY705 and pS727 play a role in this process, considering their significance in STAT3-mediated control of gene transcription. Phosphorylation of Y705 is believed to be the key event in the transcriptional activation of STAT3^9^.

One of the transcriptional targets of STAT3 that is important in oncogenesis is MUC1^10^. The promoter of MUC1 gene contains a STAT-responsive element, and STAT3 constitutively binds to it^11,12^. Inhibition of STAT3 reduced the expression of MUC1 transcription and inhibited cellular motility in breast cancer cells^12^. The MUC1 glycoprotein that is aberrantly overexpressed in several epithelial cancers and is associated with transformation, tumorigenicity, invasion, and metastasis of carcinomas is an important downstream target of STAT3^11,13,14^. Furthermore, MUC1-CT binds directly to JAK1 and STAT3 and promotes JAK1-mediated phosphorylation of STAT3. In turn, activated STAT3 induces expression of the MUC1 gene, in an auto-inductive loop. Therefore, it has been proposed that targeting STAT3 and MUC1 together may be a strategy for enhanced anti-tumor efficacy^15^.

Since STAT3-MUC1 signaling is constitutively active in high-MUC1 tumor cells, we hypothesized that high-MUC1 cells are more sensitive to STAT3-inhibition. Although STAT3 has been a target for developing cancer therapy for a while, STAT3 inhibitors have not been successful in the clinic. For example, the STAT3 inhibitor Napabucasin (BBI608) was assessed under phase III clinical trials for gastrointestinal (GI) cancers, including pancreatic cancer. However, the trial was discontinued due to futility^16^. To better understand the causes of treatment failure, the complex mechanisms of STAT3 signaling need to be elucidated. In this study, we aimed to understand the role of MUC1 in regulating differential phosphorylation of STAT3 and whether inhibiting STAT3 by Napabucasin interrupts MUC1-STAT3 interaction and downstream oncogenesis.

## Results

### STAT3 is overexpressed and correlates with poor overall survival in epithelial cancers

**Figure 1:**
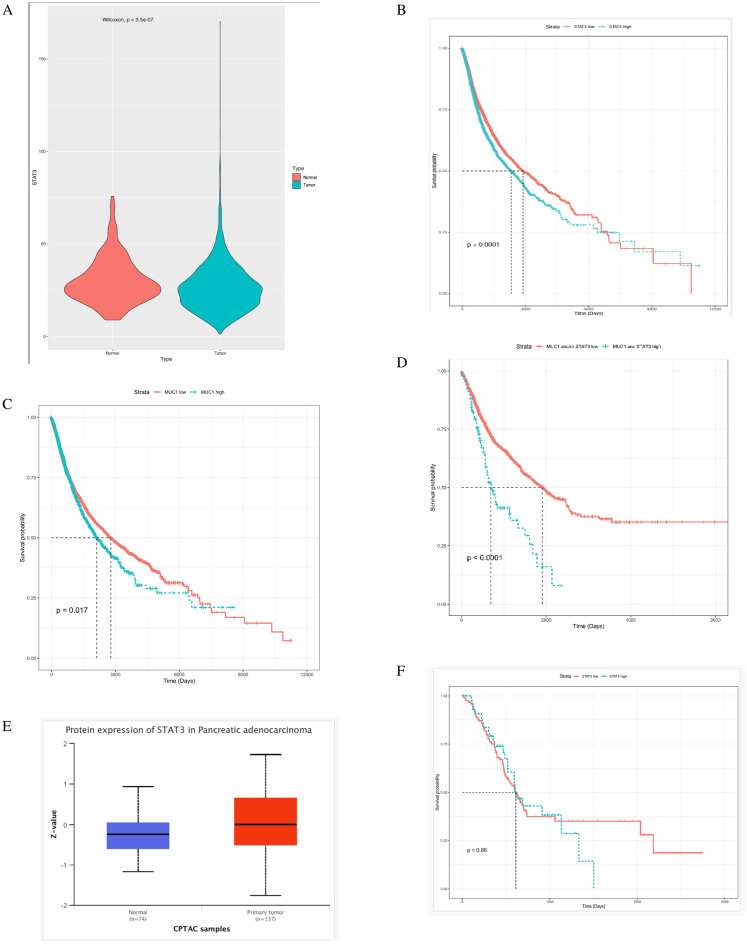
MUC1-STAT3 co-expression in epithelial cancers (**A**) Graph showing increased expression of STAT3 in epithelial tumor tissues (green) compared to normal (red). (**B**) Kaplan–Meier survival plot showing correlation of STAT3 with overall poor prognosis in epithelial cancers (red- low STAT3; green-high STAT3). (**C**) Kaplan–Meier survival plot showing correlation of MUC1 with overall poor prognosis in epithelial cancers. (**D**) Kaplan–Meier survival plot showing correlation of MUC1 and STAT3 co-expression in gastrointestinal cancers (red-low MUC1 and STAT3; green-high MUC1 and STAT3). (**E**) Expression levels of STAT3 in pancreatic ductal adenocarcinoma compared to normal from TCGA database (normal n = 74, PDA n = 137). (**F**) Kaplan–Meier survival plot showing correlation of STAT3 with overall poor prognosis in PDA (red- low STAT3; green-high STAT3).

To assess the translational significance of STAT3-MUC1 interactions in epithelial cancers, we analyzed tumor data from The Cancer Genome Atlas (TCGA) and found that STAT3 was significantly overexpressed in majority of epithelial cancers vs normal (Fig. 1A) and correlated with worse survival outcomes (Fig. 1B). MUC1 expression positively correlated with worse overall survival in gastrointestinal cancers, as previously shown (Fig. 1C)^14^. In addition, we found that STAT3 and MUC1 co-expression significantly correlated with reduced survival in gastrointestinal cancers (Fig. 1D), thus showing the amplifying oncogenic effect that MUC1 and STAT3 have together. Since we used pancreatic ductal adenocarcinoma (PDA) model for functional analyses, the expression of STAT3 in PDA and correlation with overall survival are also shown (Fig. 1E and F).

### High MUC1 expressing tumor cells are more sensitive to Napabucasin compared to low MUC1 expressing cells

Since MUC1 and STAT3 co-expression led to worse outcomes, we hypothesized that MUC1 activates STAT3 through an unknown mechanism, amplifying its oncogenic effect. Therefore, we first aimed to study the pharmacodynamics of a STAT3-inhibitor Napabucasin in low vs high MUC1 tumor cells in vitro. We used two human PDA cells lines that constitutively express high MUC1 (CFPAC and HPAFII) along with six pairs of isogenic human and murine tumor cell lines and stably transfected them with either an empty vector or full length MUC1, designated as "Neo" and "MUC1", respectively. These isogenic cells included human pancreatic cancer cells MiaPaca2 and BxPc3, murine colon cancer cell line MC38, breast cancer cell line C57MG and ovarian cancer cell line MOVCAR. In addition, we used murine pancreatic cancer cell lines KCM (that express full-length human MUC1) and KCKO (genetically null for MUC1)^17,18^.

**Figure 2:**
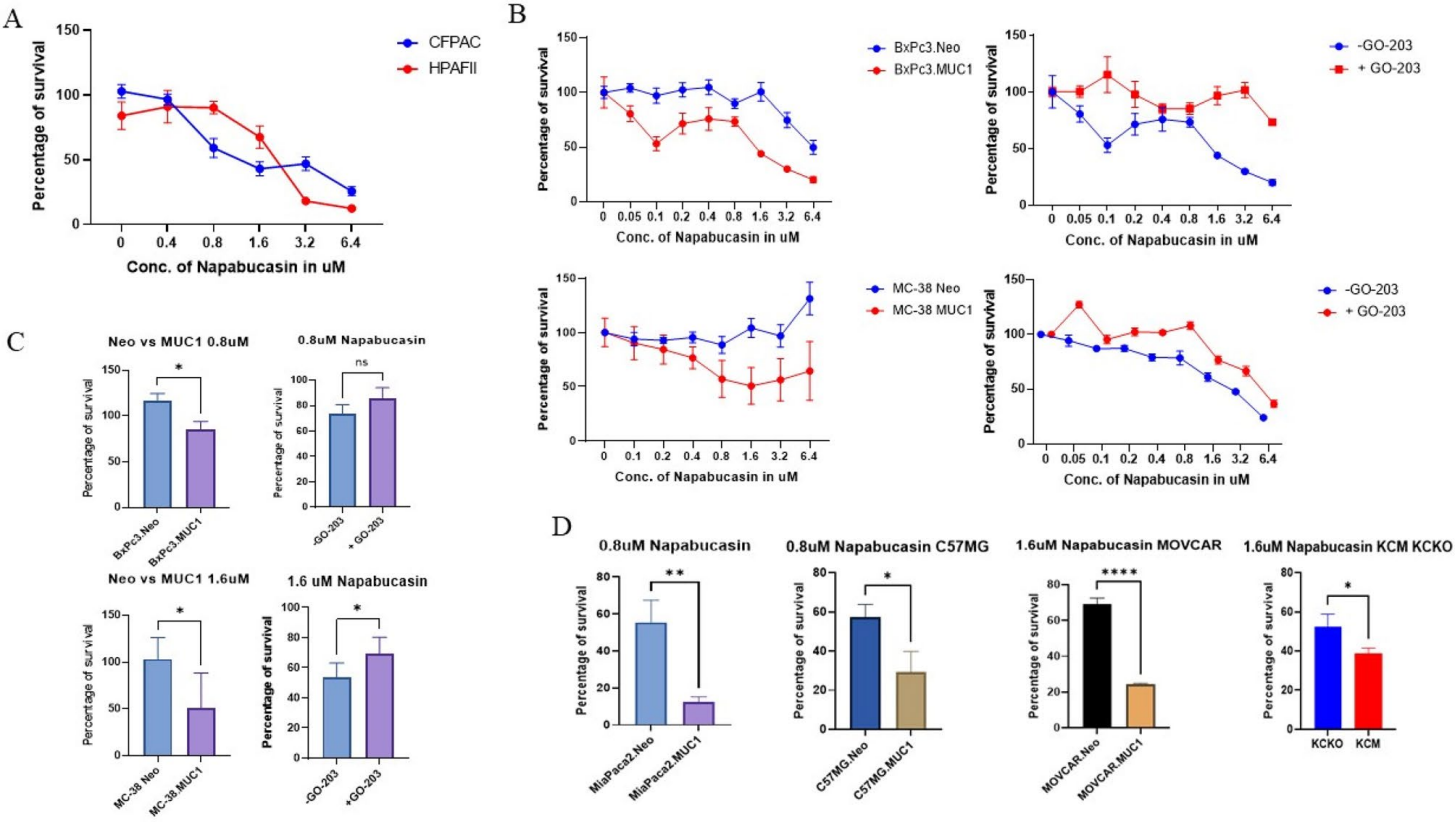
Napabucasin is more potent in high-MUC1 cells. (**A**) Survival assay showing the kill curve of Napabucasin on human PDA cell lines CFPAC and HPAFII after 48 h. (**B**) Survival assay showing the kill curve on murine cancer cells transfected with either empty vector (Neo) or full-length MUC1 with or without 1–2 h of pre-treatment with 10 μM of MUC1-homodimerization blocking peptide GO-203 followed by the indicated concentrations of Napabucasin treatment for 72 h have been shown. (Left) BxPc3.Neo and BxPc3.MUC1, BxPc3.MUC1 before and after treatment with GO-203 (top right), MC38.Neo and MC38.MUC1 (bottom left) and MC38.MUC1 before and after GO-203 treatment (bottom right) to increasing concentrations of Napabucasin. (**C**) Survival assay showing the sensitivity of (top) BxPc3.Neo and BxPc3.MUC1 and (bottom) MC38.Neo and MC38.MUC1 to 0.8 μM and 1.6 μM Napabucasin respectively after 72 h, and that of BxPc3.MUC1 and MC38.MUC1 before and after treatment with 10 μM of GO-203 (survival percentage plotted against untreated). (**D**) Survival assay showing the sensitivity of MiaPaca2.Neo and MiaPaca2.MUC1, C57MG.Neo and C57MG.MUC1, MOVCAR.Neo and MOVCAR.MUC1 and KCKO and KCM cells to 0.8 μM and 1.6 μM Napabucasin after 72 h. One-way ANOVA was used to compare difference between two groups across different doses in (**A**) and (**B**), and Student’s t-test was used to compare between two groups in (**C**) and (**D**), the graphs have been shown with S.E.M for three independent biological replicates and p value of < 0.05 was considered statistically significant.

To determine whether MUC1 plays a role in STAT3’s sensitivity to Napabucasin, we treated the panel of low and high MUC1 expressing cancer cells with increasing concentrations of Napabucasin. We found that the IC_50_ values of Napabucasin in PDA cells lines CFPAC and HPAFII (both expressing constitutively high MUC1) were similar (Fig. 1A). However, in the subsequent experiments that used isogenic cell lines, we found that the IC_50_ values of the drug were significantly lower in high MUC1 cells (BxPC3.MUC1 and MC38.MUC1) than that in their low/no MUC1 expressing counterparts (BxPC3.Neo and MC38.Neo) (Fig. 2B-left panel). Interestingly, when these cells were pretreated for 1–2 h with 10 µM of GO-203, a peptide that blocks MUC1 homodimerization and therefore MUC1 signaling, we noted a reversal in the response to the drug (Fig. 2B right panel), suggesting that MUC1 oncogenic signaling may be creating a therapeutic vulnerability toward Napabucasin. This was further confirmed in the same cell lines using a single dose (0.8 µM) of Napabucasin with and without pretreatment with GO-203 (Fig. 2C). To further test our hypothesis that high MUC1 cells are more susceptible to the anti-tumor effects of Napabucasin, we tested the effect of the drug on a few more isogenic cells lines that express high and low/no MUC1 (MiaPaca2.MUC1 and Neo; C57.MG.MUC1 and Neo; Movcar.MUC1 and Neo; and KCM and KCKO) (Fig. 2D). Additionally, Napabucasin treatment significantly reduced the tumorigenic properties of pancreatic cancer cells in vitro, namely, spheroid forming, colony forming and invasion potential (Supplementary Fig. 1). There was a dramatic effect on the tumorigenic properties of CFPAC and MiaPaca2 cells with Napabucasin, however, HPAF II being known as a resistant cell line, did not show a huge reduction in spheroid and colony forming potential. Taken together, the data convincingly showed that high expression of MUC1 in multiple tumor types sensitizes the cells to the STAT3 inhibitor Napabucasin. Furthermore, homodimerization of MUC1 CT may be necessary for inceased response to the drug. Thus, the data are in line with previous findings that MUC1 CT enhances STAT3 activation in cancer cells.

### MUC1 levels determine the phosphorylation status of STAT3

**Figure 3:**
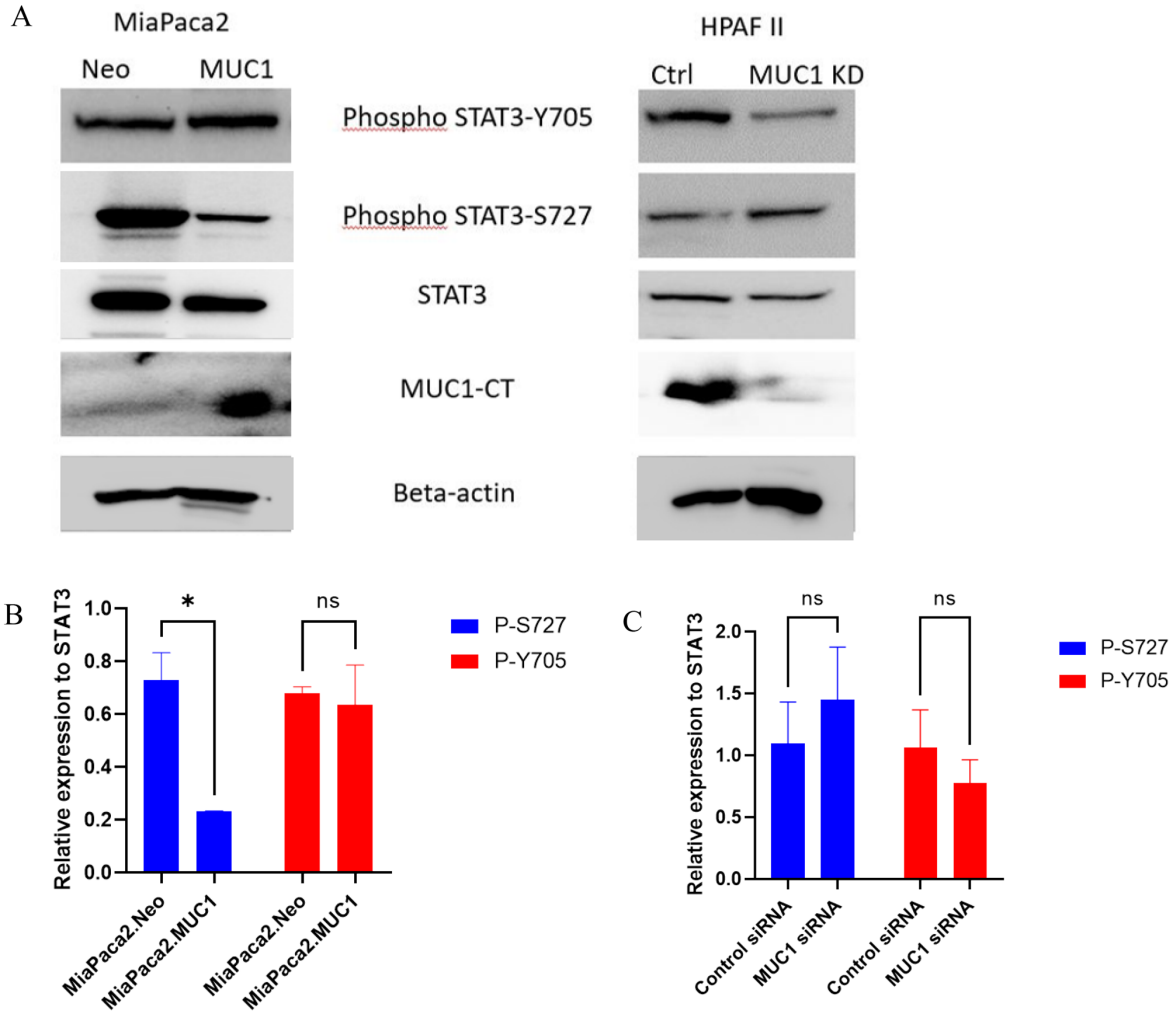
MUC1 levels determine the phosphorylation status of STAT3 in PDA cells. (**A**) Western blot showing the expression of P-STAT3 Tyrosine 705 and Serine 727, total STAT3, MUC1-CT, and β-actin on (left) MiaPaca2.Neo and MiaPaca2.MUC1 cells and (right) HPAFII with control siRNA and HPAFII with MUC1 knockdown. (**B**) Densitometric analysis of phosphorylated STAT3 S727 and Y705 in MiaPaca2.Neo and MiaPaca2.MUC1 cells (**C**) Densitometric analysis of phosphorylated STAT3 S727 and Y705 in HPAFII.control siRNA treated and HPAFII MUC1.siRNA treated cells. Two-way ANOVA was used to compare two groups and two variables, the graphs have been shown with S.E.M for at least two independent biological replicates and p value of < 0.05 was considered statistically significant.

Phosphorylation at Y705 (pY705) is believed to be essential for STAT3 transcriptional activity. Previous studies have shown that phospho-S727 regulates STAT3 activity by enhancing dephosphorylation of Y705, triggering a multistep inactivation of STAT3 by rapid dissociation of pY705-SH2 through C-terminal tail modulation^19,20^. To assess if MUC1 expression levels have any effect on differential phosphorylation of STAT3, we overexpressed full-length MUC1 in MiaPaca2 cells and knocked MUC1 down in HPAF II cells with a specific siRNA. We found that overexpression of MUC1 in MiaPaca2 cells slightly increased pY705 and significantly decreased pS727 (Fig. 3B), while knockdown of MUC1 (MUC1 KD) in HPAFII cells decreased pY705 and increased pS727, albeit not statistically significant, the trend was evident (Fig. 3C). Furthermore, we tested the same on three pairs of isogenic cell lines C57MG.Neo and MUC1, MOVCAR.Neo and MUC1 and MC38.Neo and MUC1 and found that overexpression of MUC1 significantly increased P-STAT3 Y705 and decreased P-STAT3 S727 (Supplementary Fig. 3A, C and D). These data suggest that MUC1 expression has some effect on the phosphorylation status of the Y705 and S727, and therefore plays a role in the subsequent activity of STAT3.

### Napabucasin reduces phosphorylation of STAT3 at Y705 in high-MUC1 cells

**Figure 4:**
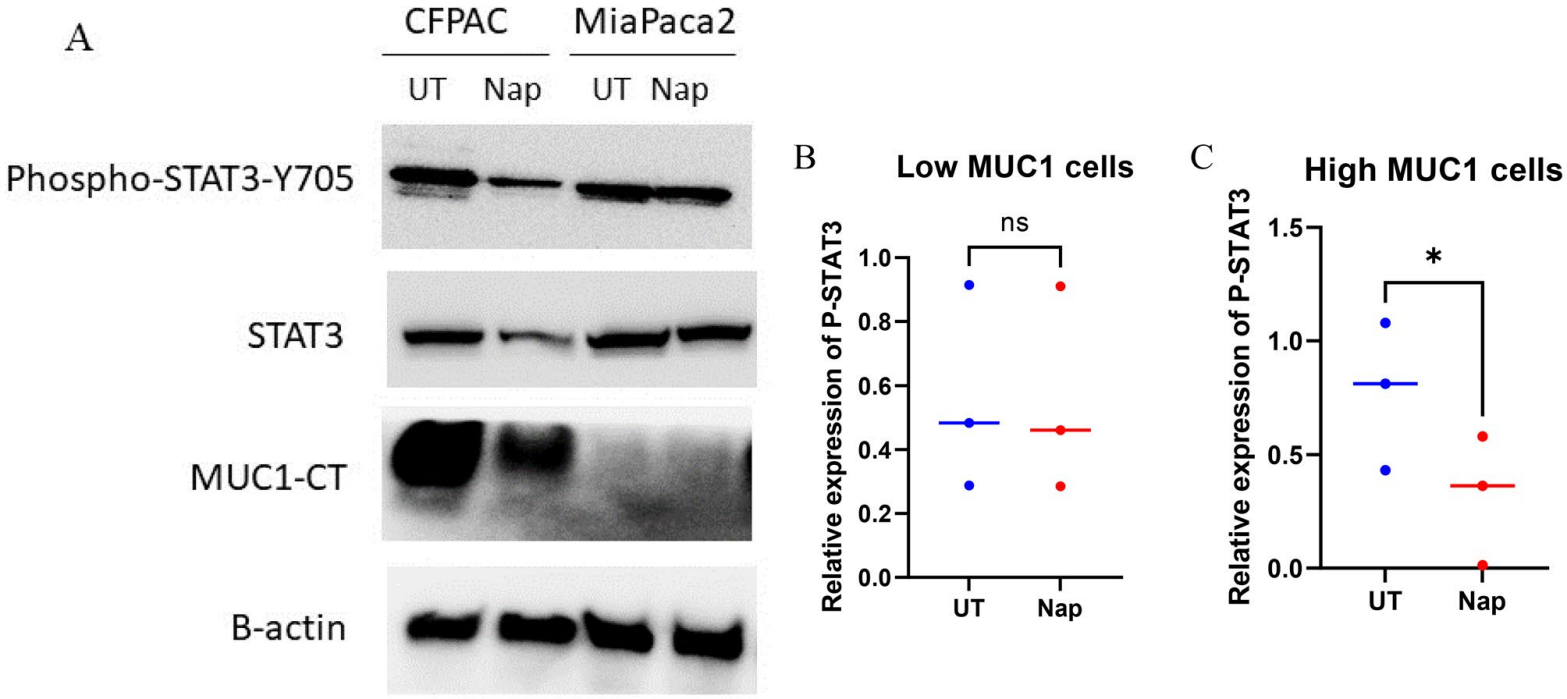
Napabucasin reduces STAT3 activity in high-MUC1 PDA cells. (**A**) Western blot showing expression of P-STAT3 Tyrosine 705, STAT3, MUC1-CT in CFPAC and MiaPaca2 cells before and after treatment with 2 μM of Napabucasin for 48 h. β-actin was used as endogenous control. (**B**) Densitometric analysis of phospho-STAT3 Y705 in low and (**C**) high MUC1 cells relative to STAT3 expression after Napabucasin treatment. Student’s t-test was used to compare two groups, graphs have been shown with S.E.M for three independent biological replicates and p value of < 0.05 was considered statistically significant.

To assess if Napabucasin has any effect on phosphorylation of STAT3 Y705 motif, and if that is affected by MUC1 expression level, we treated high and low MUC1 PDA cells (CFPAC and MiaPaca2 respectively) with 2 µM of Napabucasin for 48 h and measured the differences in phosphorylation of STAT3 at Y705, total STAT3 and MUC1 expression levels. After treatment with Napabucasin, high-MUC1 CFPAC cells had significantly lower levels of pSTAT3 at Y705, decreased STAT3 and MUC1 expression (Fig. 4A and C).

In contrast, we did not observe these effects in phosphorylation in the low MUC1 MiaPaca2 post Napabucasin treatment (Fig. 4A and B). We also observed the same trend with other isogenic pairs of cell lines, for example MC38.Neo and MC38.MUC1 and C57MG.Neo and C57MG.MUC1 (data not shown). This supports our hypothesis that in high-MUC1 PDA cells, STAT3-MUC1 interaction and signaling is constitutively activated as a survival pathway and Napabucasin inhibits this pathway.

### MUC1-STAT3 co-expression correlated to EMT and drug resistance genes in human tumor samples stratified into low and high MUC1 groups

**Figure 5:**
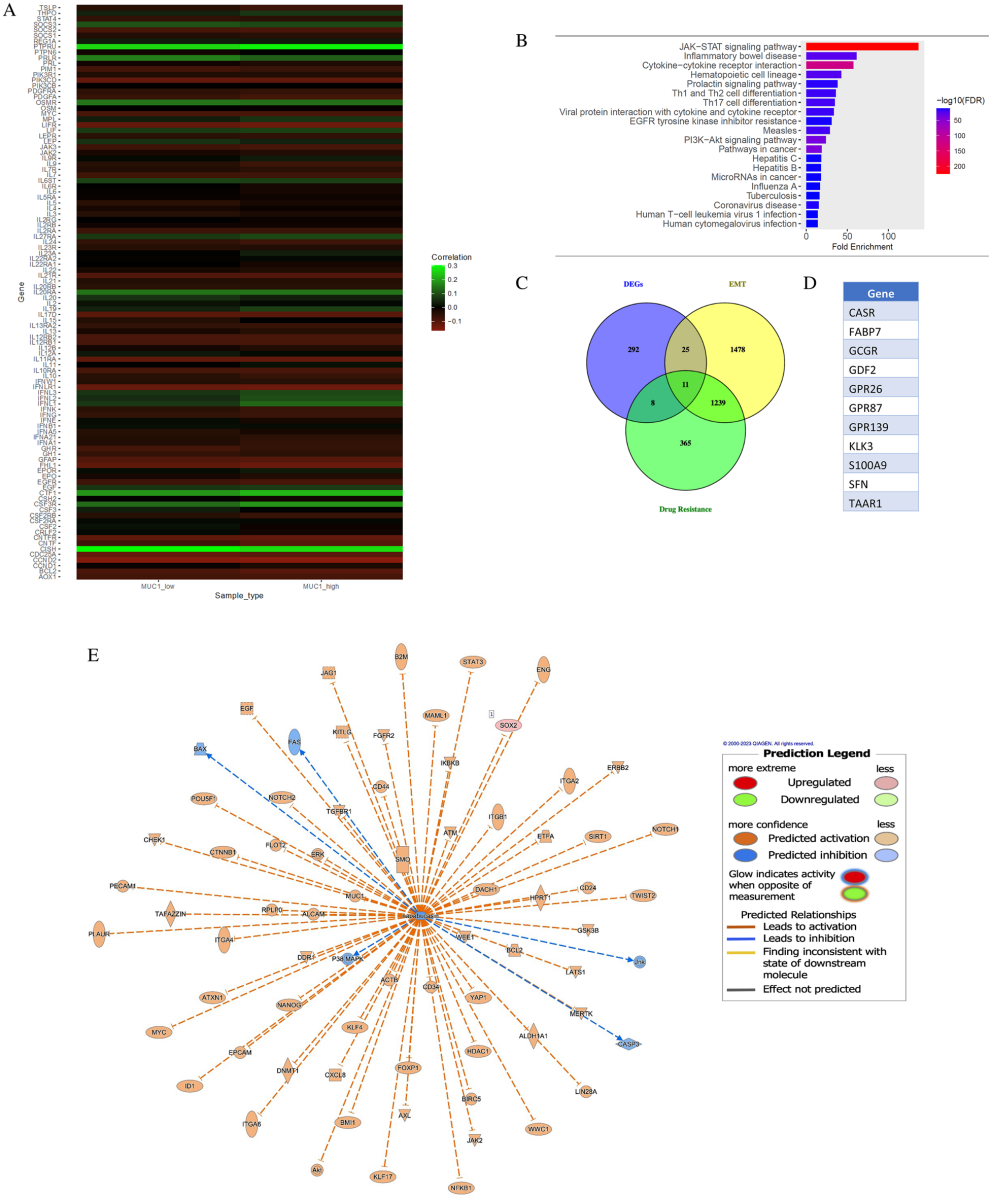
MUC1 and STAT3 co-expression is involved in EMT, drug-resistance and are part of the Napabucasin network. (**A**) Heatmap showing gene correlation of STAT3 pathway genes and MUC1 gene expression. Differential gene correlation analysis results from MUC1 low vs. high gene expression in 13,509 TCGA tumor samples were used to visualize difference in correlation with STAT3 pathway genes (103 pathway genes remained in results from filtering/analysis). (**B**) Pathway enrichment analysis with the differentially expressed genes from (**A**) using ShinyGO 0.76.3 **C.** The Venn diagram displays the common genes between DEGs from MUC1 low vs. high in BRCA, PAAD, CESC, LIHC, and OV TCGA samples with EMT and drug resistance related pathways. (**D**) List of genes common between EMT and drug resistance. (**E**) Molecular interaction network for Napabucasin overlaid with DEG results from MUC1 low vs. high in BRCA, PAAD, CESC, LIHC, and OV TCGA samples. The Napabucasin molecular interaction network displays the relationship between the drug and targeted molecules. The DEG results from MUC1 low vs. high in BRCA, PAAD, CESC, LIHC, and OV TCGA samples were overlaid on to the network to display predicted impact. In this dataset, SOX2 is observed to be significantly differentially expressed (shown in red). Line colors represent the predicted activation state of the molecule (orange represents predicted activation and blue is predicted inhibition).

To assess the clinical relevance of our data, we performed Differential Gene Correlation Analysis (DGCA) of STAT3 pathway genes with MUC1 gene expression. DGCA showed over 103 genes in the STAT3 pathway with differential correlation with MUC1 expression (Fig. 5A). Genes that showed more than two-folds difference in correlation to MUC1 were mostly from the interleukin (IL) and interferon (IFN) families (Fig. 5A). Pathway enrichment analysis of these genes showed that the top pathways altered in high vs low MUC1 samples include JAK-STAT pathway, EGFR inhibitor resistance and PI3K-Akt pathways amongst many others (Fig. 5B). Since both MUC1 and STAT3 are known to regulate Epithelial-to-Mesenchymal Transition (EMT) and drug resistance, we were interested to find out the key genes that are involved in their partnership. We overlaid the Differentially Expressed Genes (DEGs) in MUC1 high vs low and MUC1-STAT3 dual high vs low with genes in the EMT and drug resistance pathways and found 11 DEGs in MUC1 high vs low samples illustrated in a Venn diagram (Fig. 5C and D) and 13 DEGs in MUC1-STAT3 dual high vs low samples that participate in both EMT, and drug resistance (Supplementary Fig. 2A). Since high MUC1 expression and high MUC1-STAT3 co-expression correlated to drug resistance, we wanted to focus on Napabucasin resistance specifically and find genes involved in this nexus. The molecular interaction network of Napabucasin was overlaid with the DEGs in low vs high MUC1 samples (Fig. 5E) and MUC1-STAT3 dual low vs high samples from TCGA (Supplementary Fig. 2B). The molecular interaction network analysis of Napabucasin overlaid with DEGs in high vs low MUC1 showed predicted inhibition of all stemness markers that are upregulated in cancers (orange line with inhibition), namely STAT3, MUC1, ALDH1A1, Notch1, Yamanaka factors MYC, SOX2, KLF4 and more, and predicted activation of FAS, p38 MAPK, JNK, Caspase 3 and BAX that are usually downregulated (blue lines with arrowhead), out of which only SOX2 was significantly differentially expressed (Fig. 5E). None of the DEGs from the Protein–protein Interaction (PPI) analysis in MUC1-STAT3 dual low vs high samples, were significantly differentially expressed in Napabucasin-molecular interaction network. This could be due to lack of data on the molecules involved in Napabucasin signaling. Nevertheless, it emphasizes the importance of MUC1 in the Napabucasin network and highlights how it can influence potential outcomes to Napabucasin therapy. Our data corroborated with the predicted inhibition in this network showing that Napabucasin treatment reduced MUC1 levels in CFPAC cells (Fig. 4A).

## Discussion

STAT3 has been a long-standing target for developing cancer therapy, including GI cancers. Although current standard treatments for GI cancers are somewhat efficient, recurrence is still inevitable, especially in PDA^21^. Many studies have indicated that presence of pancreatic cancer stem cells (PSCs) may be a major cause of disease relapse^22^. Therefore, targeting PSCs might be a promising strategy to eradicate PDA. Given the well-documented role of overactive STAT3 signaling in maintaining stemness in PDA, the therapeutic potential of targeting this pathway should be emphasized. Multiple attempts to develop inhibitors against STAT3 pathway have been reported^23^, and a variety of STAT3 inhibitors, including chemicals, STAT3-binding peptides, and siRNA reagents, have been developed with various degrees of success^24,25^. However, the main challenges include (1) acquired resistance against tyrosine kinase inhibitors (TKIs)^26,27^, (2) success in pre-clinical settings and eventual failure in clinical trials, (3) potential toxicity due to off-target effects owing to the pleiotropic nature of cytokines such as IL-6^28,29^. Therefore, it is important to delve deeper into the mechanisms of response and resistance of STAT3 inhibitors for successful clinical outcome.

Napabucasin (BBI608) is a novel STAT3-specific inhibitor identified by Li et al.^30^. The authors revealed that Napabucasin efficiently suppressed metastasis and relapse of a variety of cancers by inhibition of STAT3-driven gene transcription. Importantly, Napabucasin treatment impaired spheroid formation of liver cancer stem cells and downregulated the expression of stemness genes such as SOX2, BMI-1, Nanog, and c-Myc. Considering the promising preclinical data of Napabucasin as both a monotherapy and in combination with conventional chemotherapeutic methods, several clinical trials have been performed^31^. Furthermore, a phase III trial of Napabucasin for refractory colorectal cancer highlighted STAT3 as an essential target for the treatment of patients with elevated pSTAT3 expression^32^. However, to improve outcomes in the clinic, it is crucial to find subpopulations of tumors that will be more sensitive to Napabucasin. In 2009, MUC1 was ranked as the second most targetable antigen to develop cancer vaccines by the National Cancer Institute^33^. Since then, a plethora of studies has shown the oncogenic role of MUC1 in increasing stemness, modulating drug resistance, as well as regulating signaling pathways in epithelial cancers^14,17,34^. MUC1 is known to correlate with poor prognosis in pancreatic cancer (https://www.proteinatlas.org/ENSG00000185499-MUC1/pathology). Since MUC1 and STAT3 were found to regulate each other’s expression in an auto-inductive loop, we hypothesized that in high-MUC1 cancer cells, the STAT3-MUC1 pathway is constitutively activated as a survival pathway and therefore, high-MUC1 cells will be more sensitive to the anti-proliferative effect of STAT3-inhibitor Napabucasin. To assess the clinical relevance of our hypothesis, we analyzed RNA sequencing data across some epithelial cancers from TCGA and found that STAT3 is overexpressed and correlates with poor overall survival in these cancer types (Fig. 1 A and B). In addition, we found that co-expression of MUC1 and STAT3 correlated to further worse overall survival in gastrointestinal cancers, indicating that MUC1 enhances the oncogenic activation of STAT3.

We confirmed that Napabucasin significantly inhibited survival of the tumor cells with high MUC1 expression at lower doses compared to low MUC1 cell lines (Fig. 2). We found that MUC1 overexpression results in increased phosphorylation of STAT3 at its activation site Y705 with a concomitant decrease in phosphorylation at its degradation site S727. Importantly, when MUC1 is knocked down, the reverse is observed (Fig. 3, Supplementary Fig. 3). These data indicate that MUC1 stabilizes STAT3 by reducing its degradation signal P-S727.

Following treatment with Napabucasin, the phosphorylation of STAT3 at Y705 and total STAT3 levels were decreased in high-MUC1 CFPAC cells but not in low-MUC1 MiaPaca2 cells (Fig. 4A). This indicates that in high-MUC1 cells, STAT3 is stabilized by MUC1 with increased phosphorylation at Y705, that maintains the STAT3-MUC1 auto-inductive loop. One study^35^ found that the underlying mechanism of downregulated STAT3 protein levels was mediated by protein synthesis inhibition induced by Napabucasin. Here, we find that Napabucasin significantly reduces the levels of STAT3 and MUC1 in high MUC1 cells and possibly impairs their crosstalk, thus breaking the auto-inductive loop.

**Figure 6:**
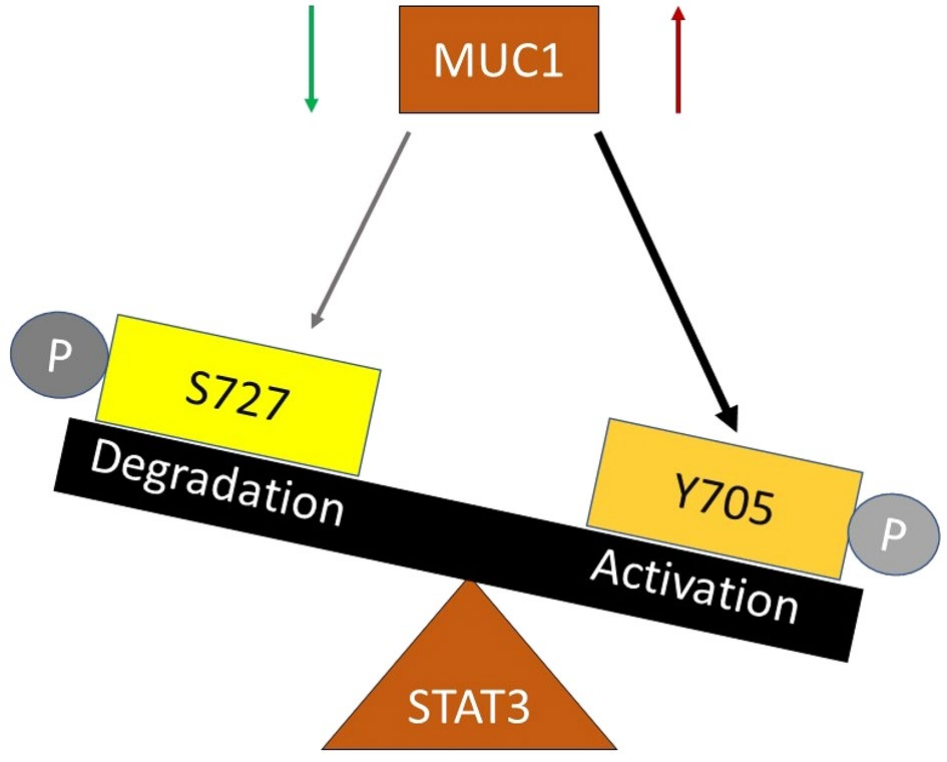
Proposed mechanism of regulation of STAT3 activity by MUC1. Overexpression of MUC1 induces phosphorylation of STAT3 at the Tyrosine 705 residue that is associated with increased activity and MUC1 downregulation induces phosphorylation of STAT3 at the Serine 727 residue that is associated with degradation.

We also identified more than 100 DEGs in the STAT3 pathway in high vs low MUC1 tumors (Fig. 5A), many of which participate in immunological functions (Fig. 5B). The role of MUC1 in tumor immunity has been studied previously^36^, however, it would be interesting to further delve into the immunological changes in context to STAT3 inhibition. The DEGs in high vs low MUC1 tumors were also associated with EMT and drug resistance (Fig. 5C,D). PPI analysis of these DEGs when overlaid with Napabucasin molecular interaction network revealed that SOX2 was significantly differentially expressed (Fig. 5E). The role of SOX2 in the Napabucasin network needs to be functionally elucidated in the future. One limitation of this study is the lack of in vivo data showing that high-MUC1 cancer cells respond better to Napabucasin treatment compared to isogenic low MUC1 cells. However, analysis of clinical data from TCGA corroborates with our hypothesis that MUC1 and STAT3 co-expression correlate to increased EMT, drug resistance and poor survival. A recent study reported that Napabucasin treatment inhibited the STAT3-MUC1 pathway in stemness-high cells and that high MUC1 status in these cells was associated with Paclitaxel resistance. It provided a new mechanism for the association between cancer stemness and drug resistance. This study also showed how combining Paclitaxel with Napabucasin could be a promising strategy to combat cancer^37^. We have shown previously^17^ and in this study that high MUC1 cells express more EMT markers, including N-Cadherin (Supplementary Fig. 3E). Another limitation of this study is the lack of validation of the hypothesis with other STAT3 inhibitors. It would be interesting to see the effect of MUC1 on the sensitivity of these cells to other STAT3 inhibitors in clinical and pre-clinical trials. To our knowledge, this is the first study to evaluate the role of MUC1 in differential phosphorylation and regulation of STAT3 in the context of Napabucasin's efficacy. Our proposed mechanism has been illustrated in Fig. 6. In our study, we reveal that Napabucasin treatment reduced activation of STAT3 and MUC1, and therefore is more potent against high-MUC1 cells. Overall, our results support the potential use of Napabucasin as an efficacious anti-tumor therapeutic agent with a probability of better outcome in high-MUC1 tumor sub-populations, singly or in combination with other therapeutic agents.

## Materials and methods

### TCGA data analysis

#### TCGA gene expression analysis

RNA-sequencing data from 7,572 epithelial cancer samples available from TCGA was analyzed and STAT3 expression values in normal and tumor samples were plotted. The types of cancers include breast, lung, endometrium, kidney, head-neck, thyroid, prostate, colon, stomach, bladder, liver, cervix, pancreas, esophagus, adrenal, and gallbladder cancer. Plot was created using ggplot2 (version 3.3.5) package in R (version 3.6.3).

#### Gene correlation analysis

Tumor samples from TCGA cancer projects were split into MUC1 low/high group based on the MUC1 gene expression relative to the average MUC1 gene expression in normal samples. Datasets were filtered to remove the lowest expressed genes by the dispersion measure. Differential gene correlation analysis was performed on these two groups using DGCA (Differential Gene Correlation Analysis) in R (version 3.6.3). Gene correlation analysis was run, using the Benjamini–Hochberg p-adjustment measure. STAT3 pathway genes were selected and their correlation values with MUC1 were visualized in a heatmap, using ggplot2 (version 3.3.5) package in R.

#### Molecular interaction network analysis

The drug network figure represents Napabucasin, and associated molecules overlaid with IPA predictions on activation states. The Molecule Activity Predictor feature in IPA overlaid DEG results from MUC1 low vs. high in BRCA, PAAD, CESC, LIHC, and OV TCGA samples on to the network to display predicted impact. The SOX2 gene, in red to show increased expression, is a DEG that is associated with Napabucasin. Other molecules are colored orange for predicted activation or blue for prediction inhibition activity. The relationship arrows were generated to match the predicted activation states.

#### Survival analysis

Survival analysis for STAT3 and overall survival in 13,509 cancer samples were computed using the Kaplan–Meier estimate and plots were made using ggplot2 (3.3.5) package in R (3.6.3). The average expression for STAT3 in normal samples was used to determine if tumor samples were low or high in STAT3 expression.

Survival analyses for MUC1/STAT3 expression and overall survival in 2,055 gastric cancer type samples were computed using the Kaplan–Meier estimate and plots were made using ggplot2 (3.3.5) package in R (3.6.3). The average expression for MUC1/STAT3 in normal samples was used to determine if tumor samples were low or high in MUC1/STAT3 expression.

### Cell lines and culture

Human PDA cell lines (CFPAC, HPAF-II and MiaPaca2) and murine cancer cell lines MC38 (colon), C57MG (breast), MOVCAR (ovarian) were obtained from American Type Culture Collection and KCM and KCKO (pancreatic) cells were generated as described^17,18^ and cultured as instructed. Cell lines were maintained in Dulbecco’s Modified Eagle Medium (DMEM; Gibco). All media was supplemented with 10% fetal bovine serum (FBS; Gibco or Hyclone), 3.4 mM l-glutamine, 90 units (U) per ml penicillin, 90 μg/ml streptomycin, and 1% non-essential amino acids (Cellgro). Cells were kept in a 5% CO_2_ atmosphere at 37℃. MUC1 WT sequence was cloned into the pLNCX.1 vector consisting of the neomycin resistance gene (neo) and confirmed by DNA sequencing. Neo cells had the empty vector with the G418 resistance gene (neo) and MUC1 cells had the full length MUC1 gene and G418 resistance gene (neo). All cells with Neo and MUC1 were generated by transfection with Lipofectamine 3000 (Thermo Fisher) according to the manufacturer's protocol and maintained in medium containing Geneticin (G418; Invitrogen, Carlsbad, CA, USA)^17^. Every passage of Neo or MUC1 transfected cells were maintained in a final concentration of 150 μg/ml of the antibiotic G418 (50 mg/ml) (Thermo Fisher) to ensure positive selection. HPAFII cells were serum-starved for 24 h and then treated with control siRNA from Life Technologies or MUC1 siRNA from Perkin Horizon according to the respective manufacturer’s protocol using Lipofectamine RNAiMAX Transfection Reagent (Thermo Fisher Scientific) for 72 h. For all experiments, cell lines were passed no more than 10 times.

### MTT assay and addition of MUC1 blocking peptide

5,000 cells were plated in 96 well plates and allowed to grow overnight. Next day, the cells were treated either with PBS or increasing concentrations of Napabucasin in triplicates for 48–72 h. Then 20 μl of MTT solution (5 mg/ml) was added to each well and incubated for 3–4 h at 37 °C. Following that, the media with MTT was removed and 200 μl of DMSO was added to each well to dissolve the formazan crystals for 10 min and the O.D. was measured with a plate reader (Multiskan, Thermo Fisher) at 560 nm. For blocking MUC1 signaling, the peptide GO-203 was added to the cells 1–2 h before treatment with Napabucasin.

### Spheroid formation assay

Non-adherent U-bottom 96-well plates (Nunc, Thermo Fisher) were seeded with 5000 cells and centrifuged at 1000xg for 10 min. These cells were treated with 1 μM of Napabucasin, and the health of cells and size and integrity of the spheroids were monitored, and pictures were taken with an inverted microscope at 4X after 72 h.

### Colony formation assay

500–1000 cancer cells were plated in a 6-well tissue culture plate and allowed to adhere overnight. Next day, the cells were treated with increasing concentrations of Napabucasin (0.1, 0.2, 0.4, 0.8 and 1.6 μM) for 7–14 days (depending on the doubling time of each cell line). PBS was used as the control. After 7–14 days, the media was removed, colonies were washed with PBS and fixed with 3:1 solution of Methanol: Acetic acid for 5 min, followed by staining with 0.5\% (w/v) of Crystal Violet in Methanol for 15 min. Then the colonies were washed under running tap water, images were taken, and colonies were counted manually. Colonies consisting of > 25 cells were considered. The number of colonies in Napabucasin treated wells were calculated as a percentage of colonies in the PBS treated wells and plotted as a kill curve in GraphPad Prism. A p value of < 0.05 was considered significant.

### Invasion assay

Cells were serum starved for 18 h before plating for the invasion assay. 50,000 CFPAC, MiaPaca2 and HPAFII cells were plated over transwell inserts (Sarstedt) precoated with diluted Matrigel (1:1) in serum free media, with 1 μM of Napabucasin. The cells were allowed to invade through the Matrigel coating for 72 h towards the serum-containing medium in the bottom chamber. After 72 h, only the control wells were swabbed with a cotton swab, followed by staining of all inserts with 5\% crystal violet. The excess stain was washed off and the inserts were allowed to dry. The membrane was cut and dipped in 10\% acetic acid for 10 min to elute the dye, which was read by a Spectrophotometer at 560 nm. Percent invasion was calculated as (O.D. of Napabucasin treated sample / O.D. of PBS treated sample) X 100.

### Treatment with napabucasin and western blotting

The cell lines used were MiaPaca2. Neo, MiaPaca2. MUC1, CFPAC, HPAFII. control siRNA, and HPAFII. MUC1siRNA. Cells were treated with either 2 μM Napabucasin (Selleckchem, USA) or the vehicle (phosphate buffer saline) for 48 h. Cell lysates were prepared and western blotting performed as previously described^34^. Membranes were blocked with commercial blocking buffer (Thermo Fisher) for 30 min at room temperature and incubated with primary antibodies overnight at 4 °C. The antibodies used were: Armenian hamster monoclonal anti-human MUC1 cytoplasmic tail (CT2) antibody (1:500). MUC1 CT antibody CT2 was originally generated at Mayo Clinic and purchased from Neomarkers, Inc. (Portsmouth, NH)^38^. CT2 antibody recognizes the last 17 amino acids (SSLSYNTPAVAATSANL) of the cytoplasmic tail (CT) of human MUC1. Membranes were also probed with the following antibodies from Cell Signaling Technology (1:1,000), p-STAT3 (Y705), total STAT3, β-actin, and from ABclonal (1:1000) p-STAT3 (S727) and β-actin. Densitometric analysis was conducted using the ImageJ software. First, each density unit for the particular protein was normalized to their respective β-actin density and then represented as phospho/total.

### Equipment and settings

For Western blot, Biorad Chemidoc was used to acquire images of blots. Some membranes were cut before hybridization with primary antibodies and therefore the full membrane image is the size of the cut membrane and sometimes the edges of the membranes will not be clearly visible since Chemidoc images have a light background. Microsoft PowerPoint was used to crop the images to fit the layout of the manuscript.

### Statistical analysis

Differences between groups were examined using unpaired two-tailed Student’s t-tests, one-way and two-way ANOVAs. Statistical comparisons were made using GraphPad Prism 9.0. p-values of < 0.05 were considered statistically significant (*p < 0.05; **p < 0.01; ***p < 0.001; ****p < 0.0001).

### Supplementary Information

Supplementary Figures.

Supplementary Information.

## Data Availability

All of the RNA sequencing data analyzed was downloaded from the Genomics Data Commons Portal: https://portal.gdc.cancer.gov.
