# Supplementary material for: Molecular crosstalk between MUC1 and STAT3 influences the anti-proliferative effect of Napabucasin in epithelial cancers
Source: Sci Rep. 2024 Feb 7;14:3178. doi: 10.1038/s41598-024-53549-4 (PMC10850135; doi:10.1038/s41598-024-53549-4)
Supplement: Supplementary file 1 — Supplementary Figures. [file 41598_2024_53549_MOESM1_ESM.pdf]

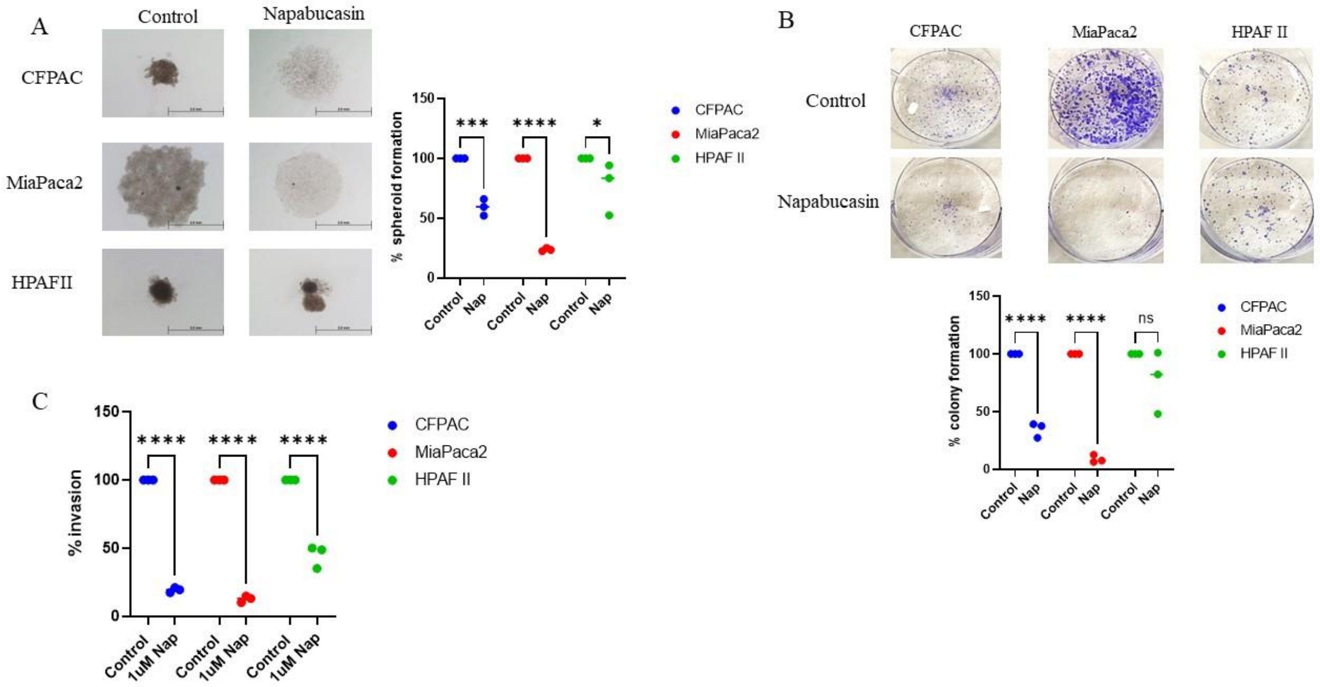

Supplementary Figure 1. Effects of Napabucasin on the tumorigenic properties of pancreatic cancer cells. Cells were treated with 1uM of Napabucasin for 48-72 hours and effects on **A.** spheroid forming potential, **B.** colony forming potential and **C.** Invasion potential were analyzed. The control was considered as 100%, data was analyzed with two-way ANOVA and shown with S.E.M. for three independent replicates and a p value of <0.05 was considered significant.

A

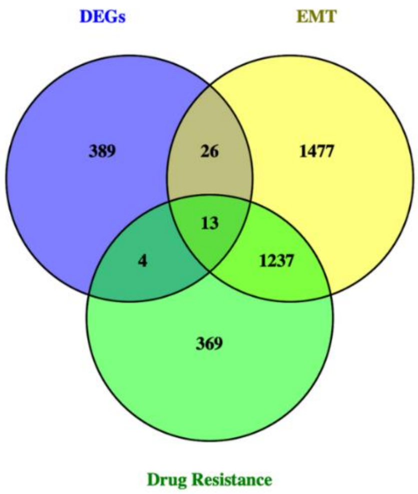

B

Gene
BCYRN1
CASR
CCKBR
FABP7
FFAR1
GPR12
GPR50
GPR139
IGF2
KLK1
OPRK1
PCDH10
PRKCG

Supplementary Figure 2. A. DEGs from MUC1/STAT3 low vs. MUC1/STAT3 high in BRCA, PAAD, CESC, LIHC, AND OV TCGA samples. The Venn diagram represents the common genes involved with MUC1/STAT3 low vs. MUC1/STAT3 high DEGs and the EMT (epithelial-mesenchymal transition) and drug resistance pathway genes. **B.** Genes from multiple IPA pathways related to EMT and drug resistance were included in each group. Intersection of DEGs from MUC1/STAT3 low vs. MUC1/STAT3 high in BRCA, PAAD, CESC, LIHC, and OV TCGA samples and genes in the EMT and drug resistance pathways.

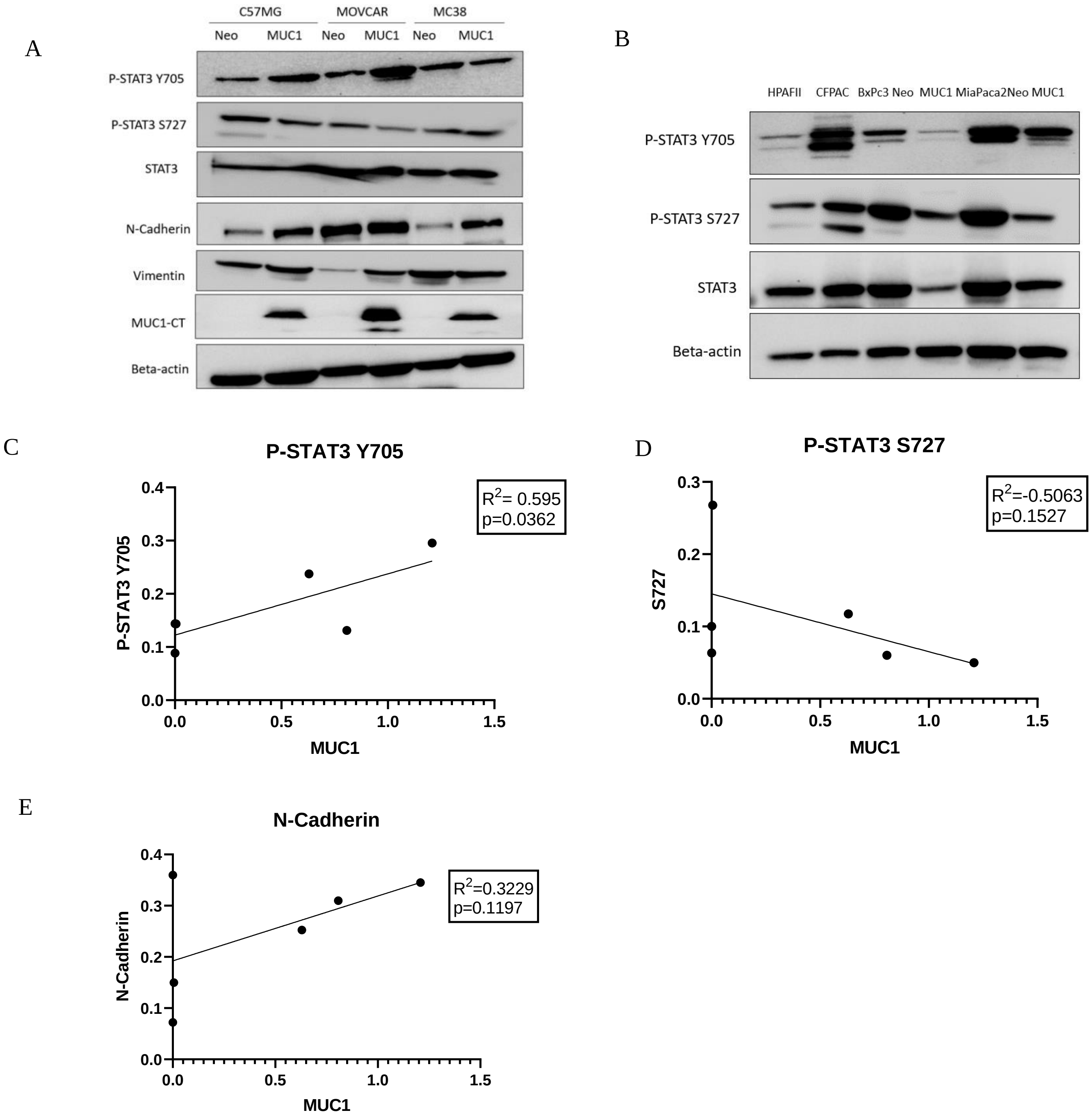

Supplementary Figure 3. Western blot showing expression levels of PSTAT3 Y705. S727, STAT3 in **A.** Mouse isogenic cell lines with or without MUC1 overexpression, **B.** human cell lines with low or high MUC1. Densitometric analysis of **C.** P-STAT3 Y705, **D.** P-STAT3 S272 and **E.** EMT marker N-Cadherin in all cell lines as a function of MUC1 expression, the correlation coefficients and p values are shown.
