# Supplementary material for: Molecular crosstalk between MUC1 and STAT3 influences the anti-proliferative effect of Napabucasin in epithelial cancers
Source: Sci Rep. 2024 Feb 7;14:3178. doi: 10.1038/s41598-024-53549-4 (PMC10850135; doi:10.1038/s41598-024-53549-4)
Supplement: Supplementary file 2 — Supplementary Information. [file 41598_2024_53549_MOESM2_ESM.docx]

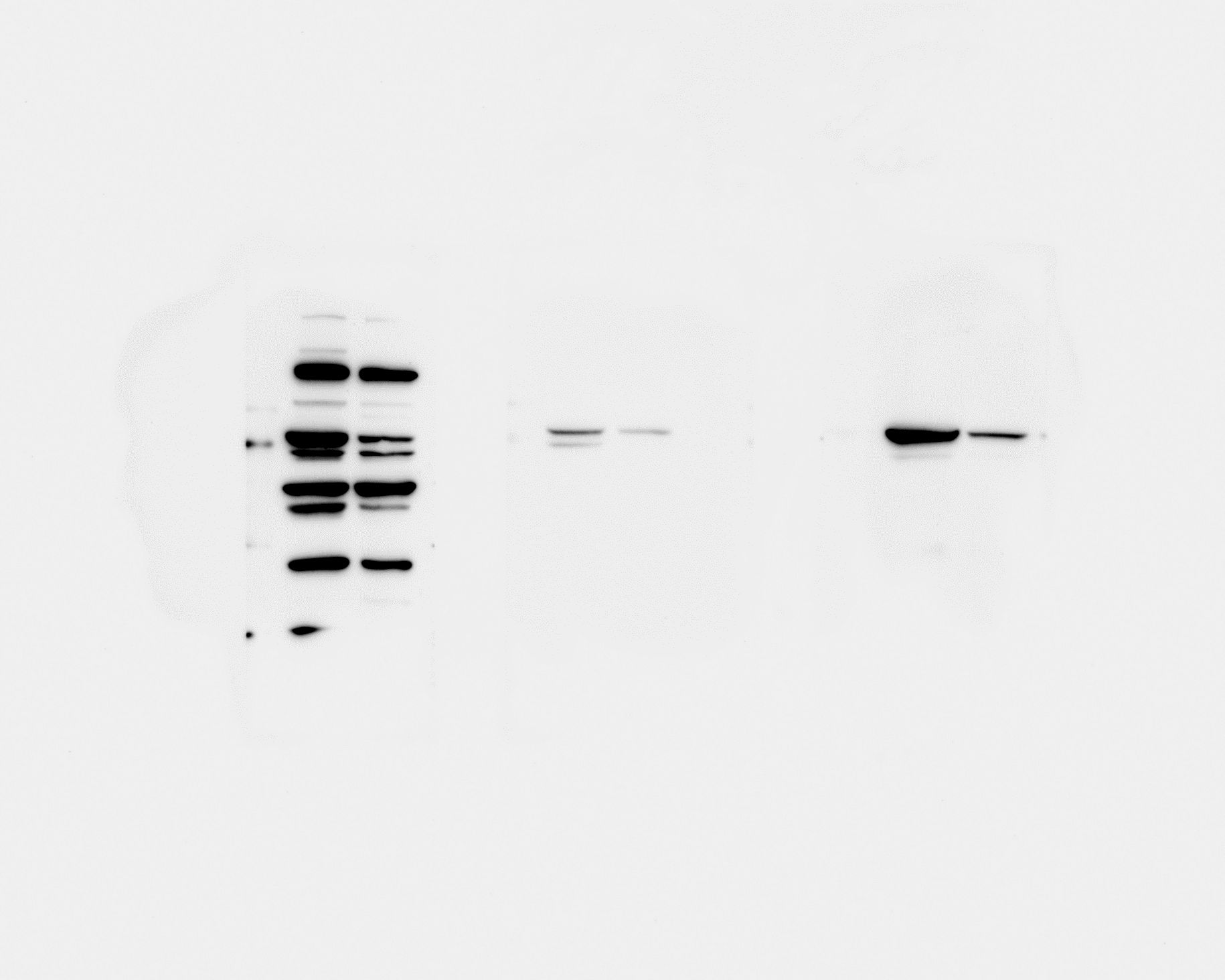


80 kDa

80 kDa

P-STAT3

S727

Neo MUC1

STAT3

Neo MUC1

Figure 3. WB for MiaPaca2.Neo and MUC1


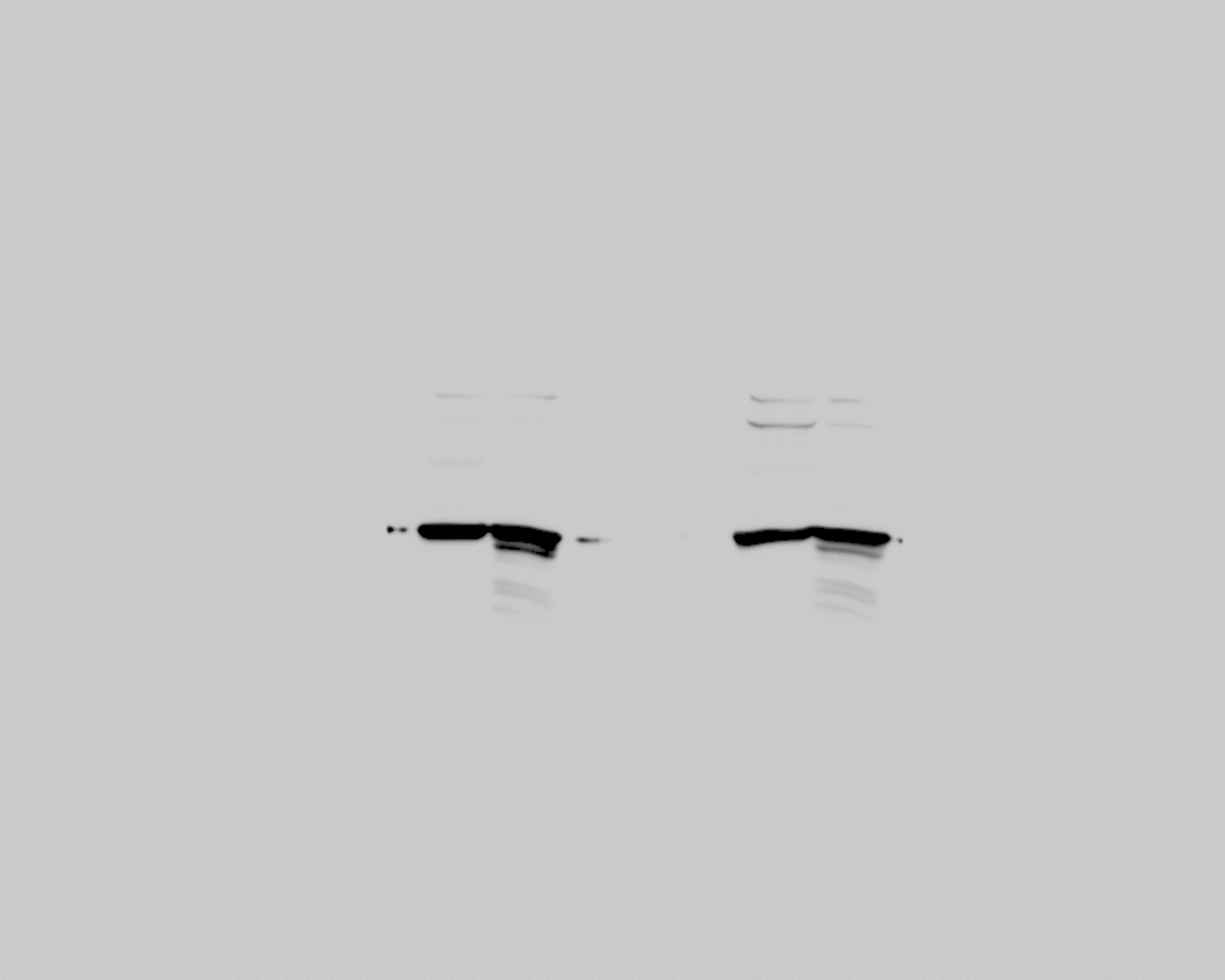


45 kDa

Beta-actin

Beta-actin

Neo MUC1

Neo MUC1

Figure 3. WB for MiaPaca2.Neo and MUC1


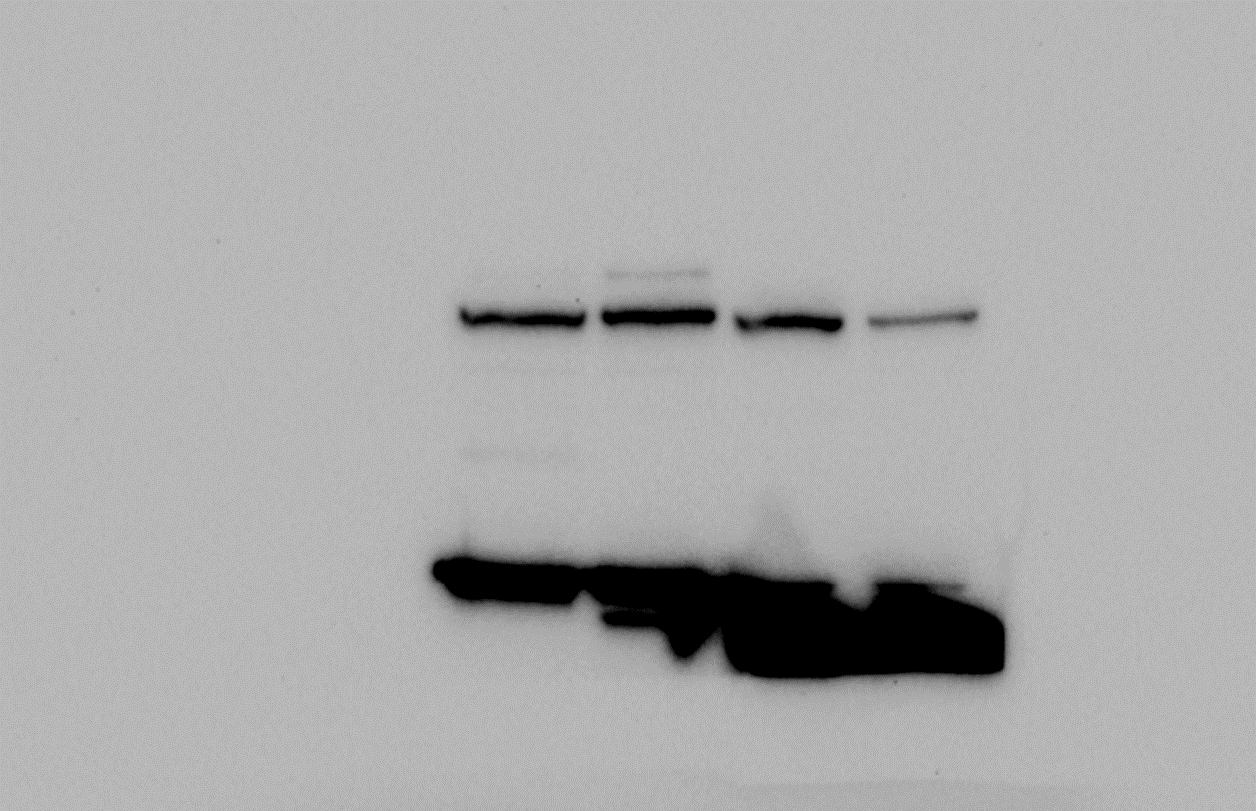


80 kDa

P-STAT3 Y705

Neo MUC1 Ctrl siRNA MUC1 siRNA

MiaPaca2 HPAFII

Figure 3. WB for MiaPaca2.Neo and MUC1 and HPAFII control siRNA and MUC1 siRNA.


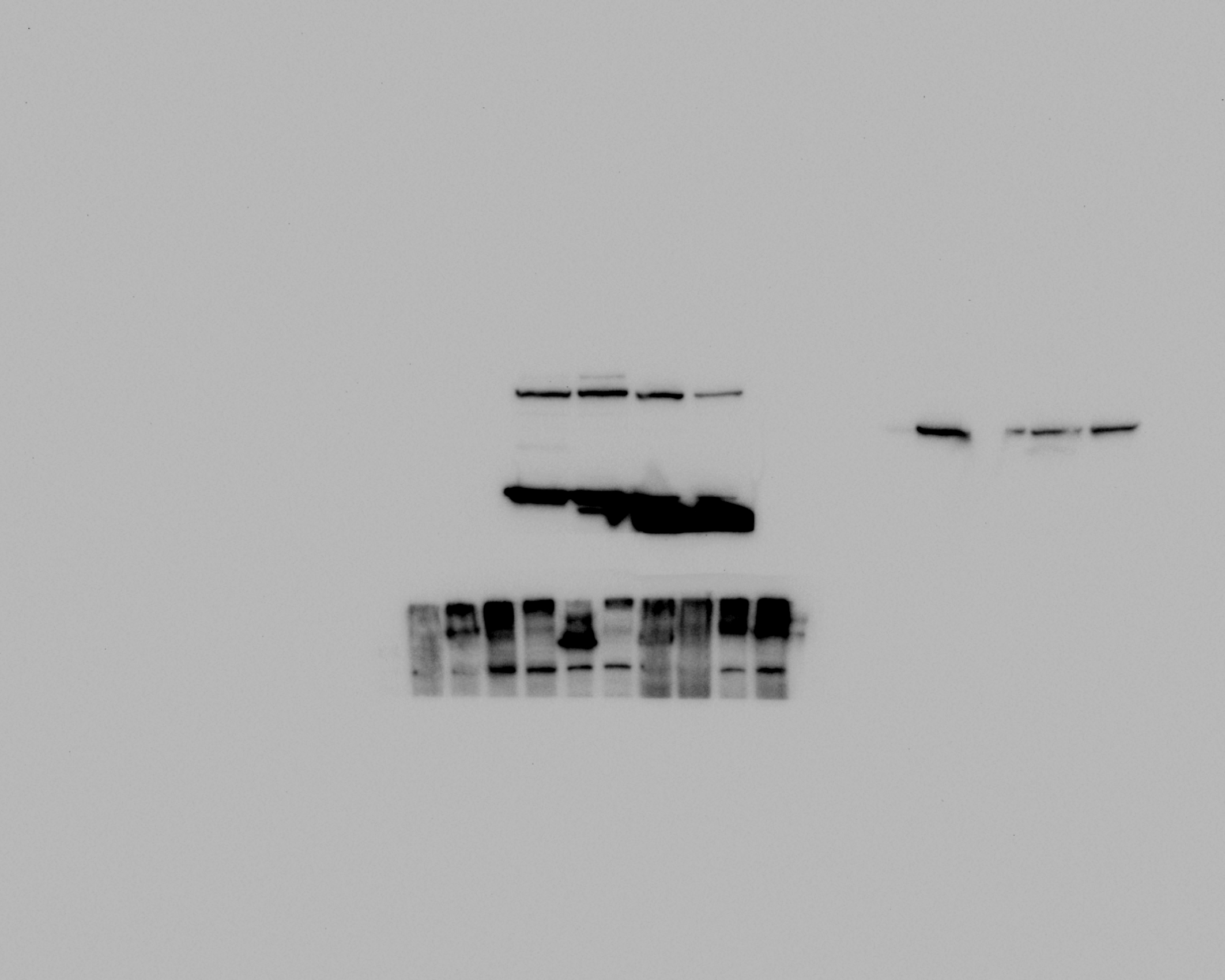


80 kDa

P-STAT3 S727

MiaPaca2 HPAFII

Neo MUC1 Ctrl siRNA MUC1 siRNA

Figure 3. WB on MiaPaca2.Neo and MUC1 and HPAFII control siRNA and MUC1 siRNA.


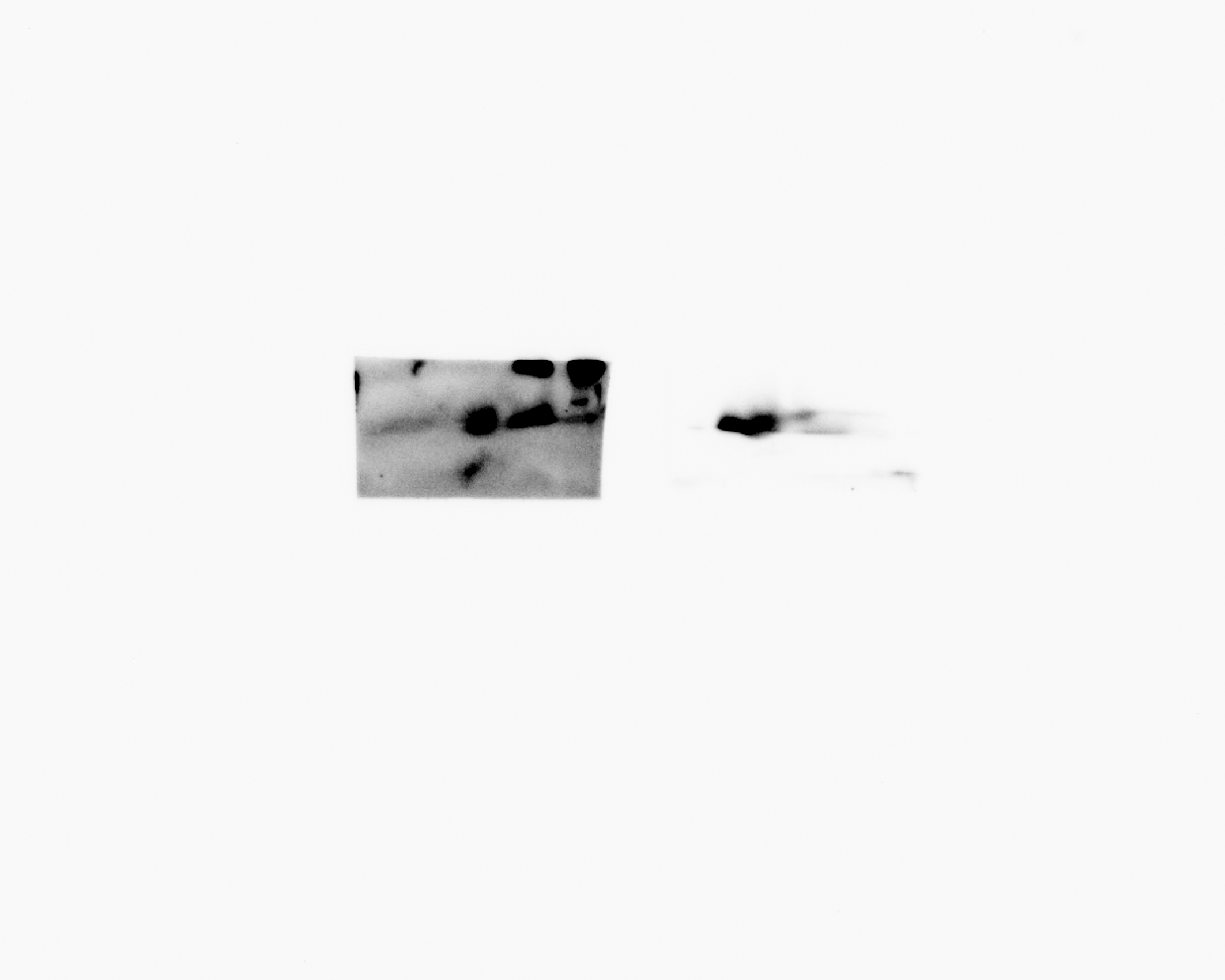


25 kDa

MUC1-CT

MiaPaca2.Neo MUC1

Figure 3. WB on MiaPaca2.Neo and MUC1.


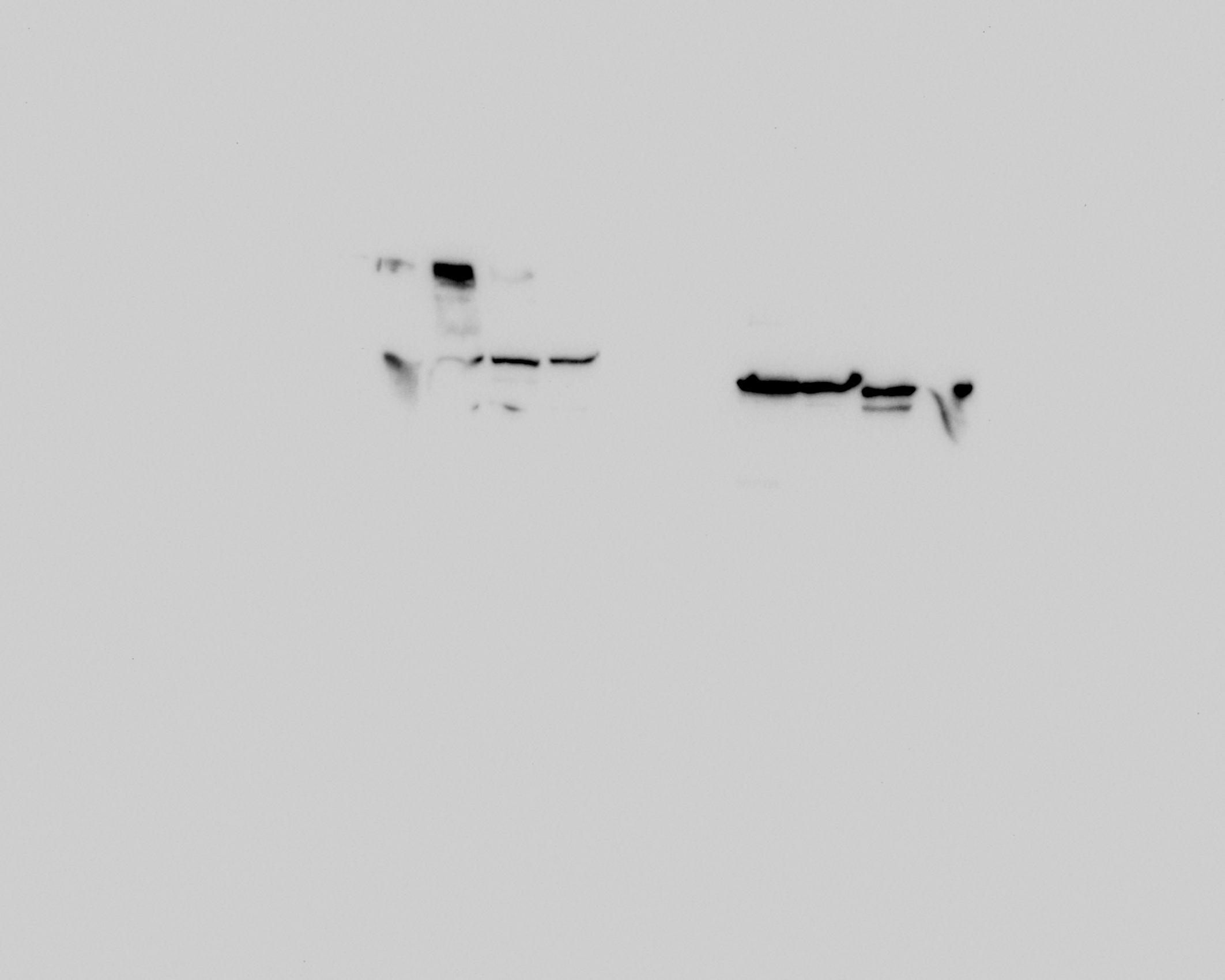


80 kDa

STAT3

HPAFII

Ctrl siRNA MUC1 siRNA

Figure 3. WB on HPAFII control siRNA and MUC1 siRNA.


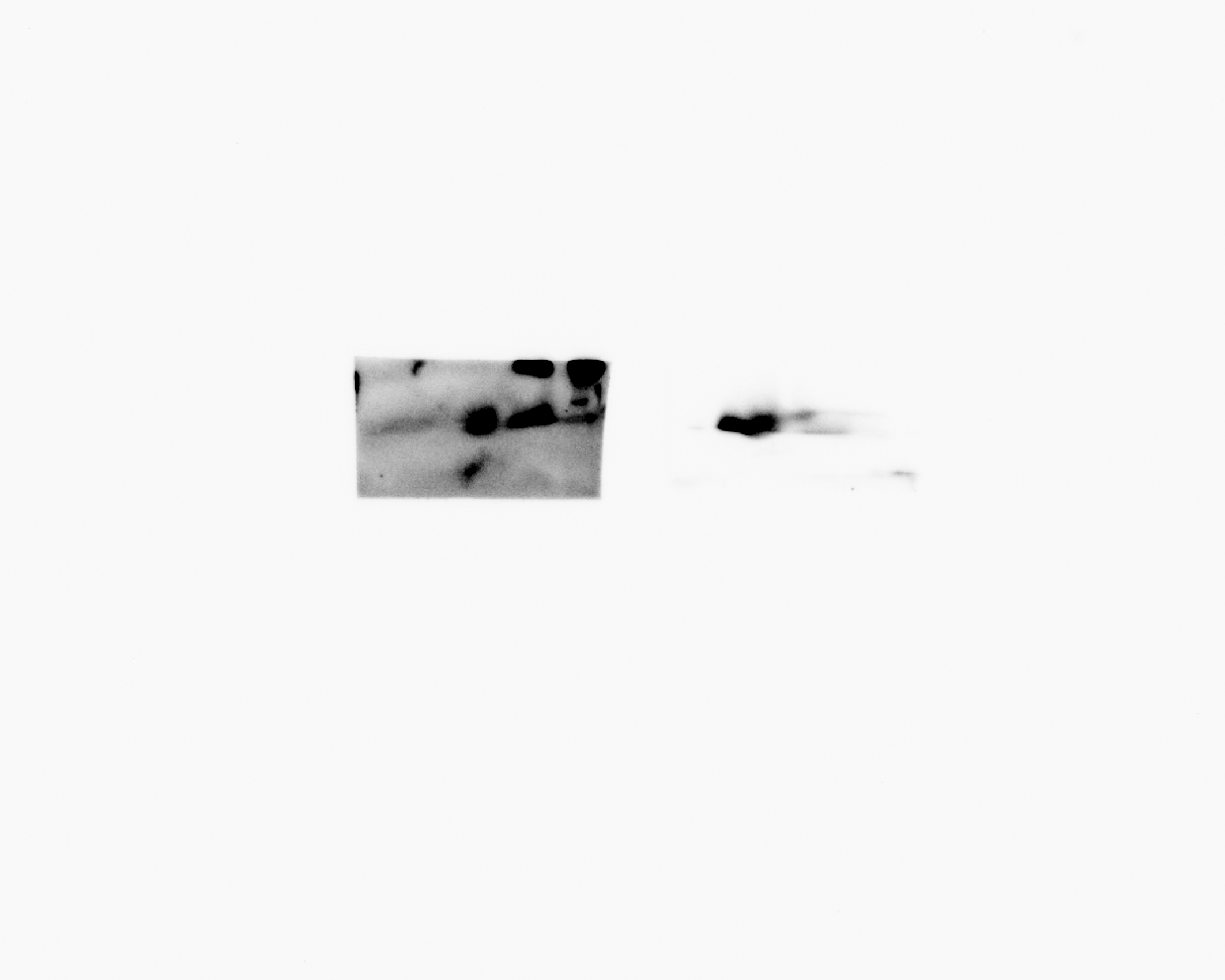


25 kDa

MUC1-CT

HPAFII

Ctrl siRNA MUC1 siRNA

Figure 3. WB on HPAFII control siRNA and MUC1 siRNA.


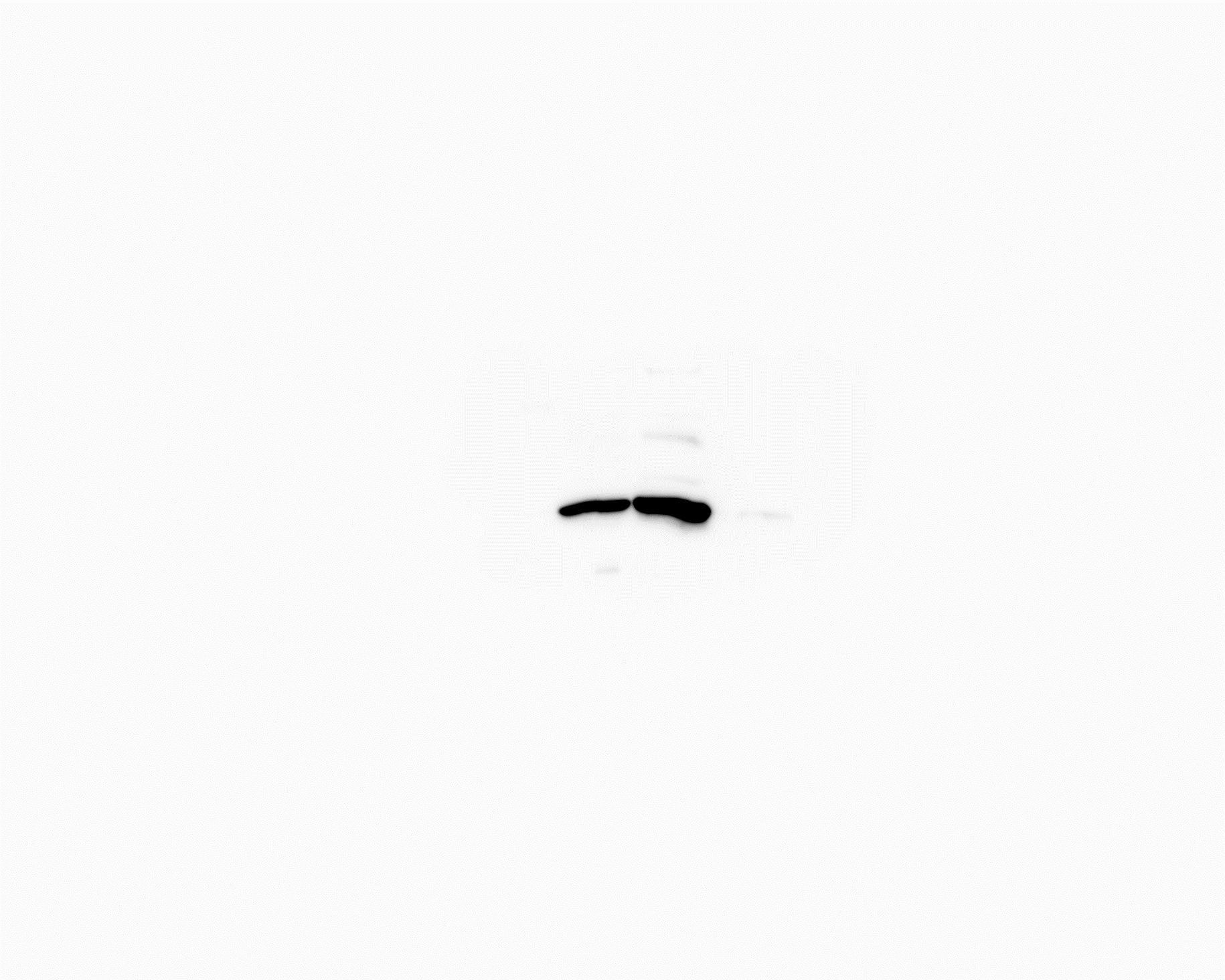


45 kDa

beta-actin

Ctrl siRNA MUC1 siRNA

Figure 3. WB on HPAF II Ctrl siRNA and MUC1 KD.


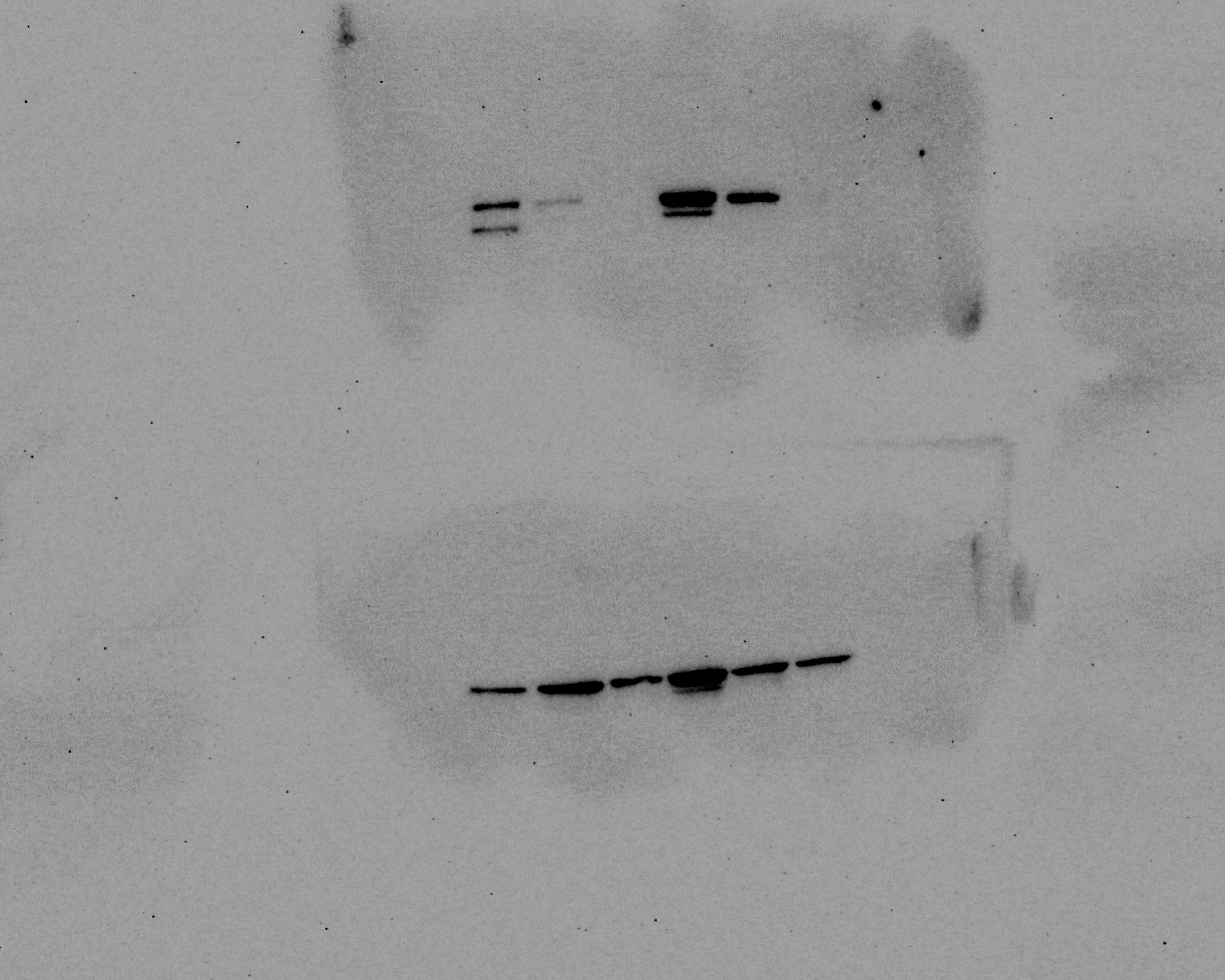


80 kDa

P-STAT3 Y705

Neo MUC1 Neo MUC1 Neo MUC1

C57MG MOVCAR MC38

Supplementary Figure 3A. WB on mouse cell lines.


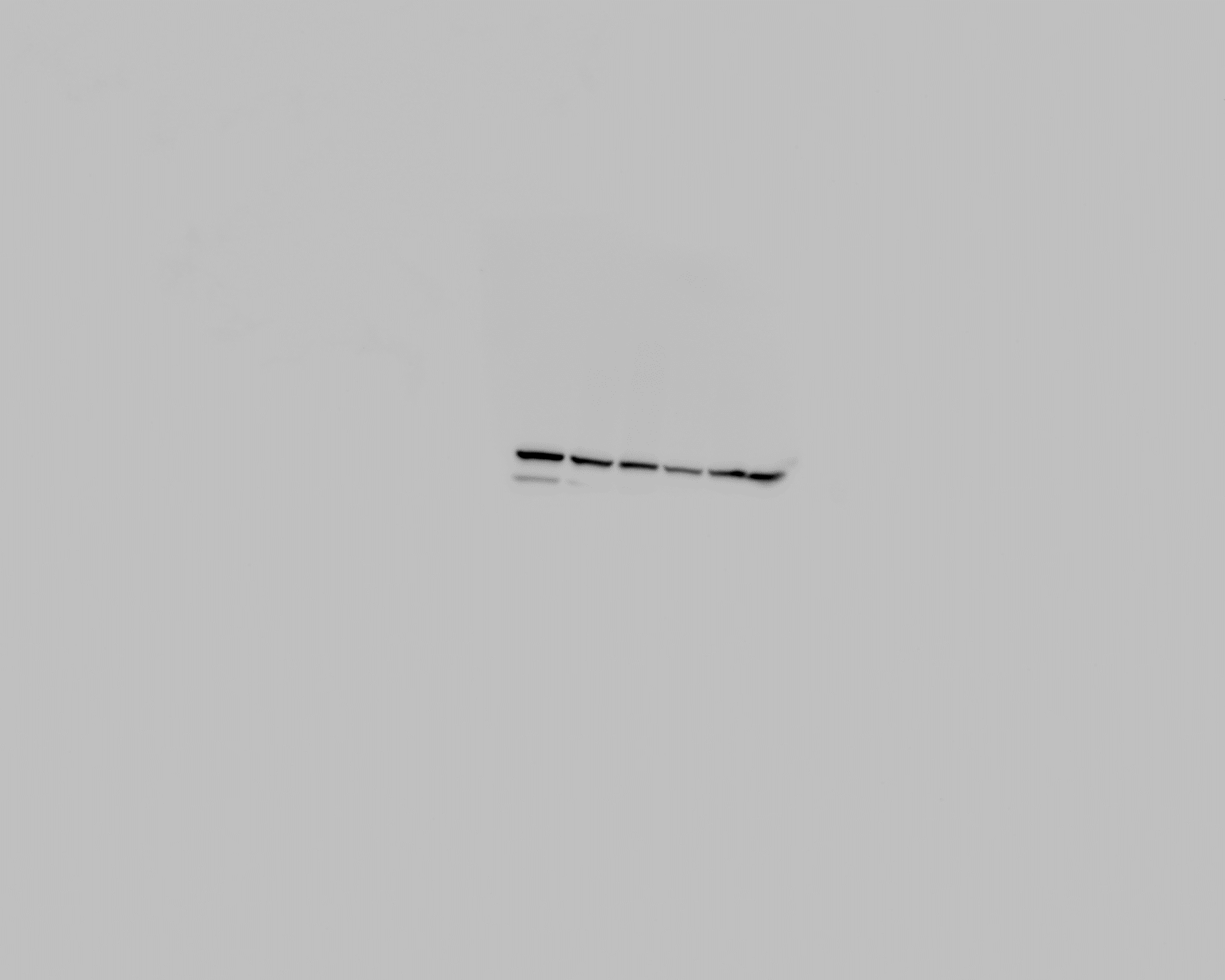


80 kDa

P-STAT3 S727

Neo MUC1 Neo MUC1 Neo MUC1

C57MG MOVCAR MC38

Supplementary Figure 3A. WB on mouse cell lines.


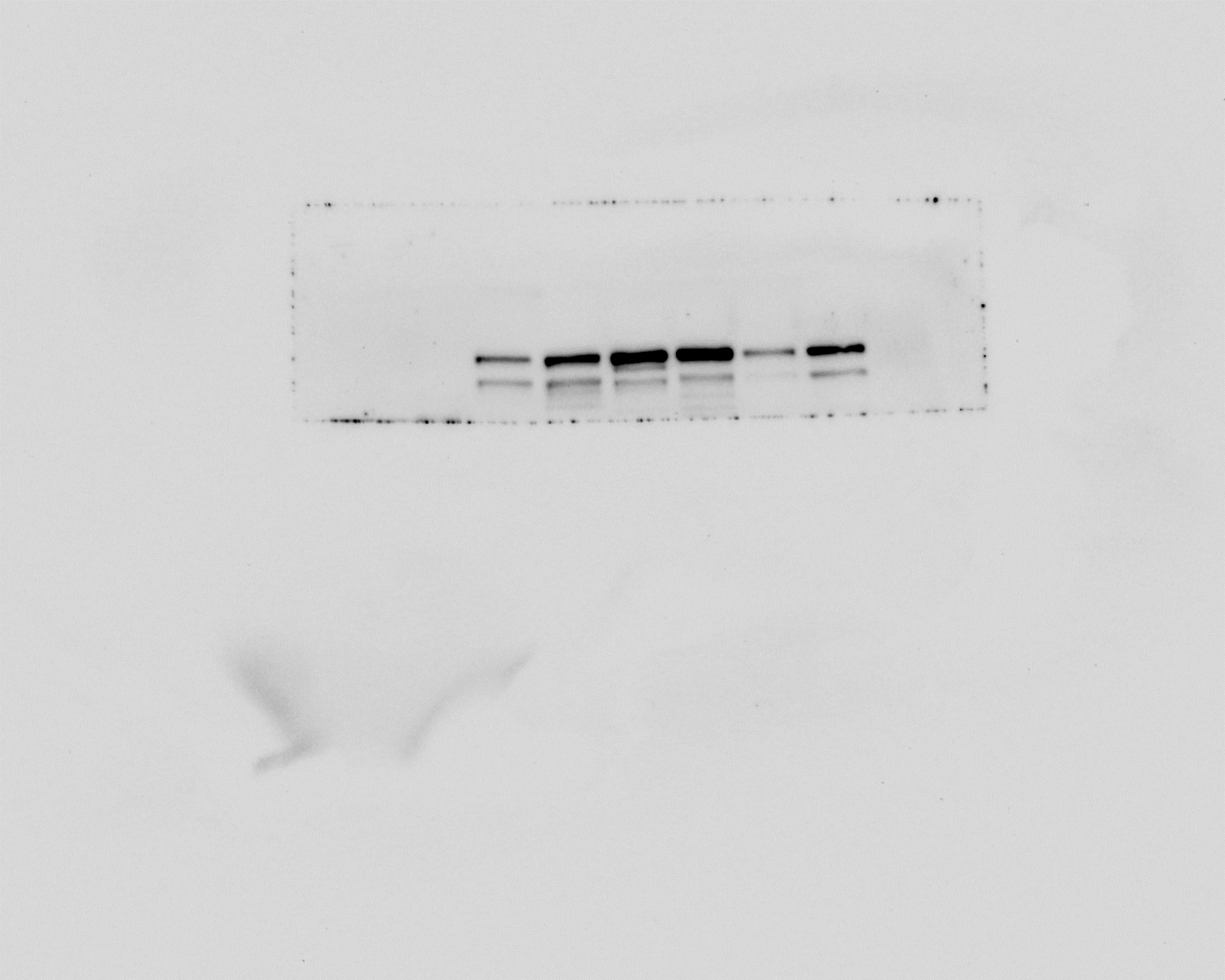


140 kDa

N-Cadherin

Neo MUC1 Neo MUC1 Neo MUC1

C57MG MOVCAR MC38

Supplementary Figure 3A. WB on mouse cell lines.


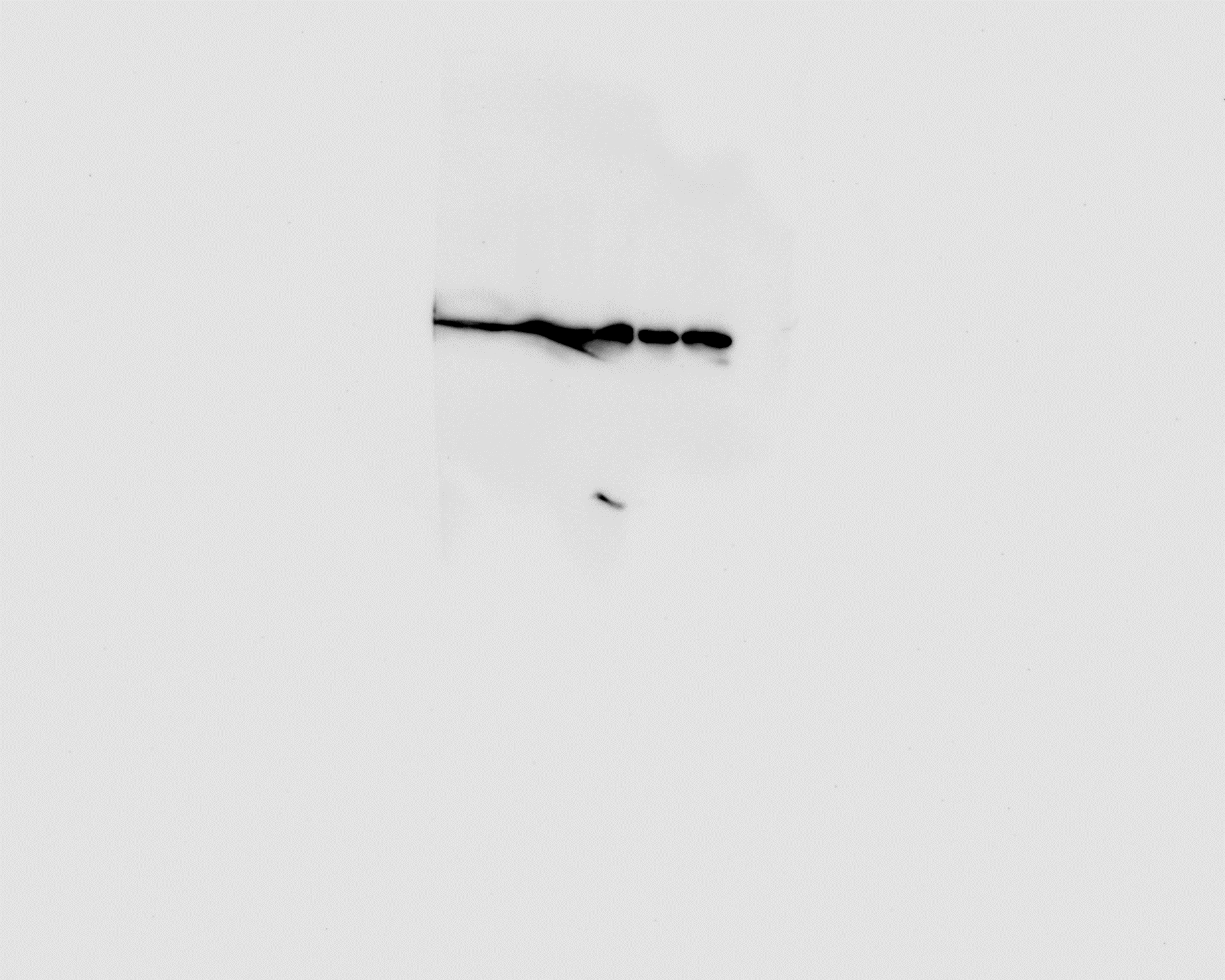


80 kDa

STAT3

Neo MUC1 Neo MUC1 Neo MUC1

C57MG MOVCAR MC38

Supplementary Figure 3A. WB on mouse cell lines.


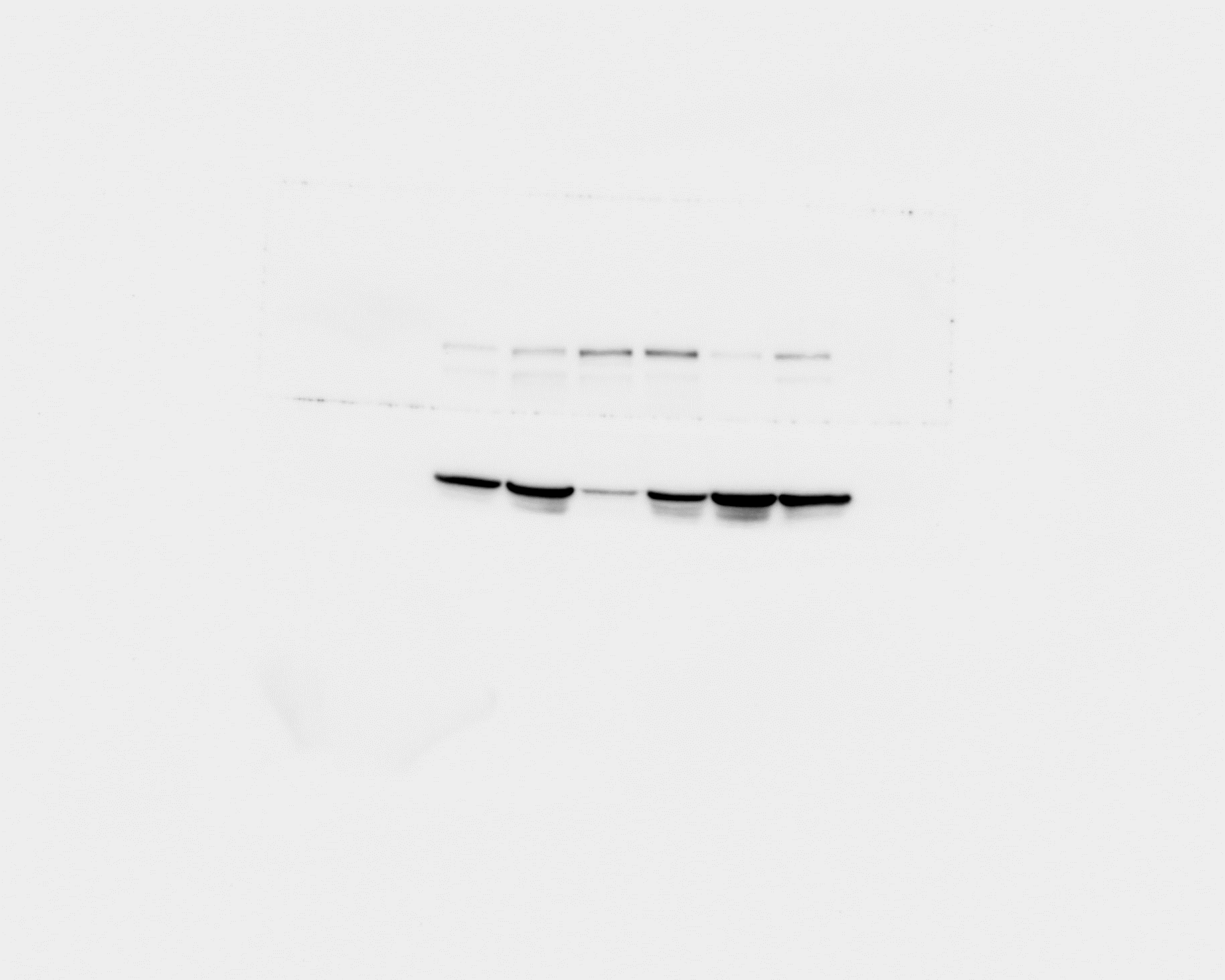


C57MG MOVCAR MC38

57 kDa

Vimentin

Neo MUC1 Neo MUC1 Neo MUC1

Supplementary Figure 3A. WB on mouse cell lines.


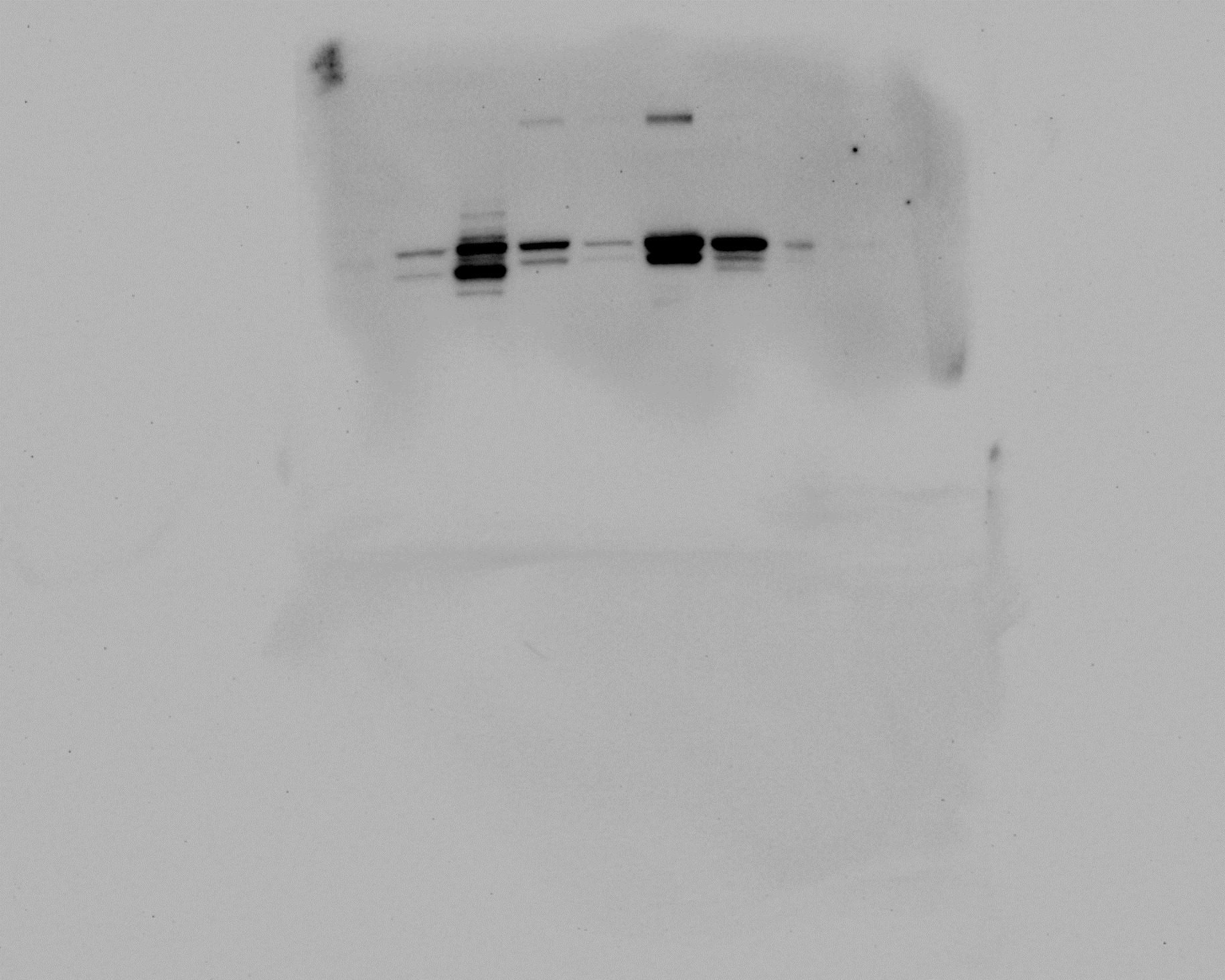


80 kDa

P-STAT3 Y705

HPAFII CFPAC BxPc3.Neo MUC1 MiaPaca2.

Neo MUC1

Supplementary Figure 3B. WB on human cell lines.


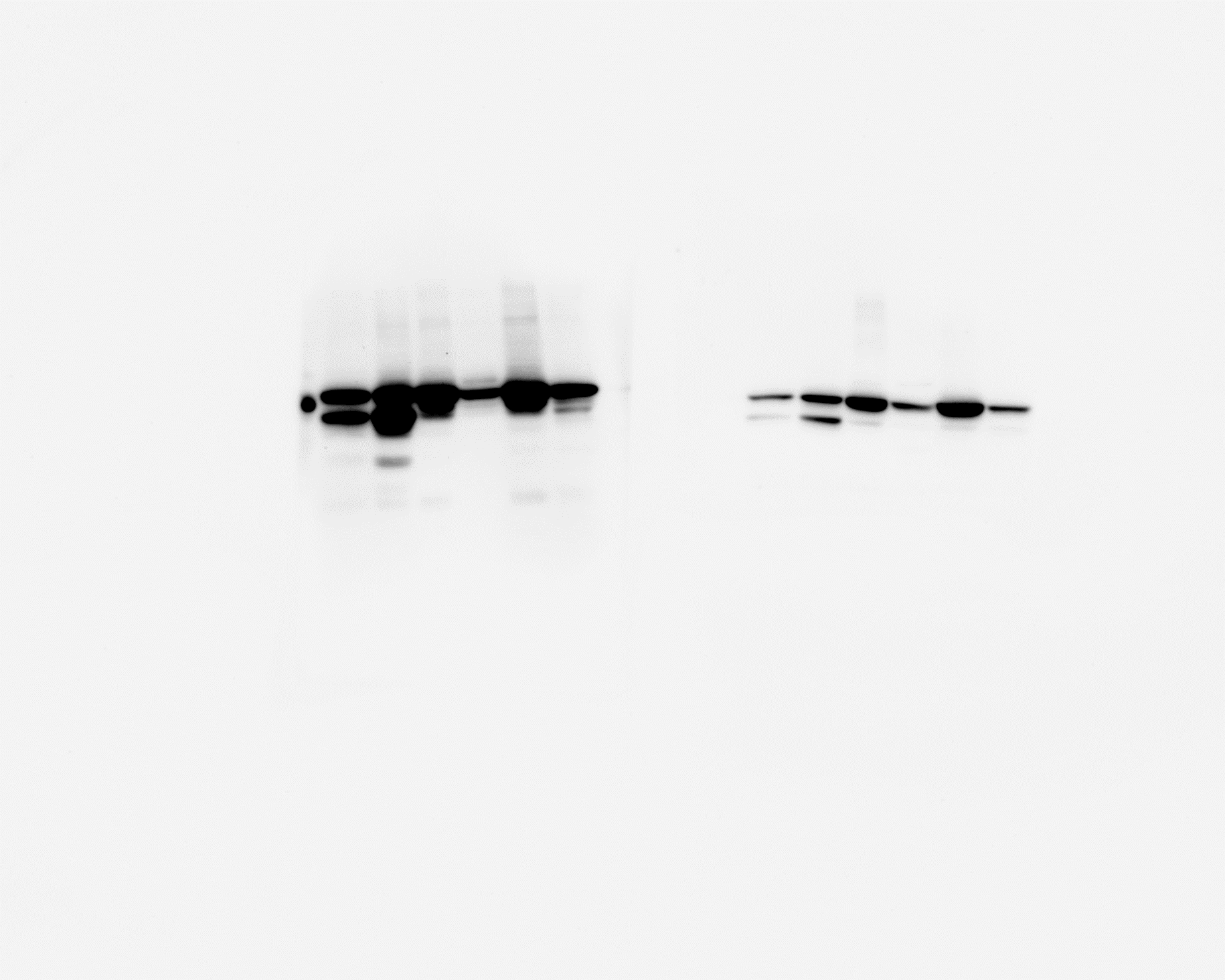


80 kDa

P-STAT3 S727

HPAFII CFPAC BxPc3. MiaPaca2.

Neo MUC1 Neo MUC1

HPAFII CFPAC BxPc3. MiaPaca2.

Neo MUC1 Neo MUC1

STAT-3

Supplementary Figure 3B. WB on human cell lines.


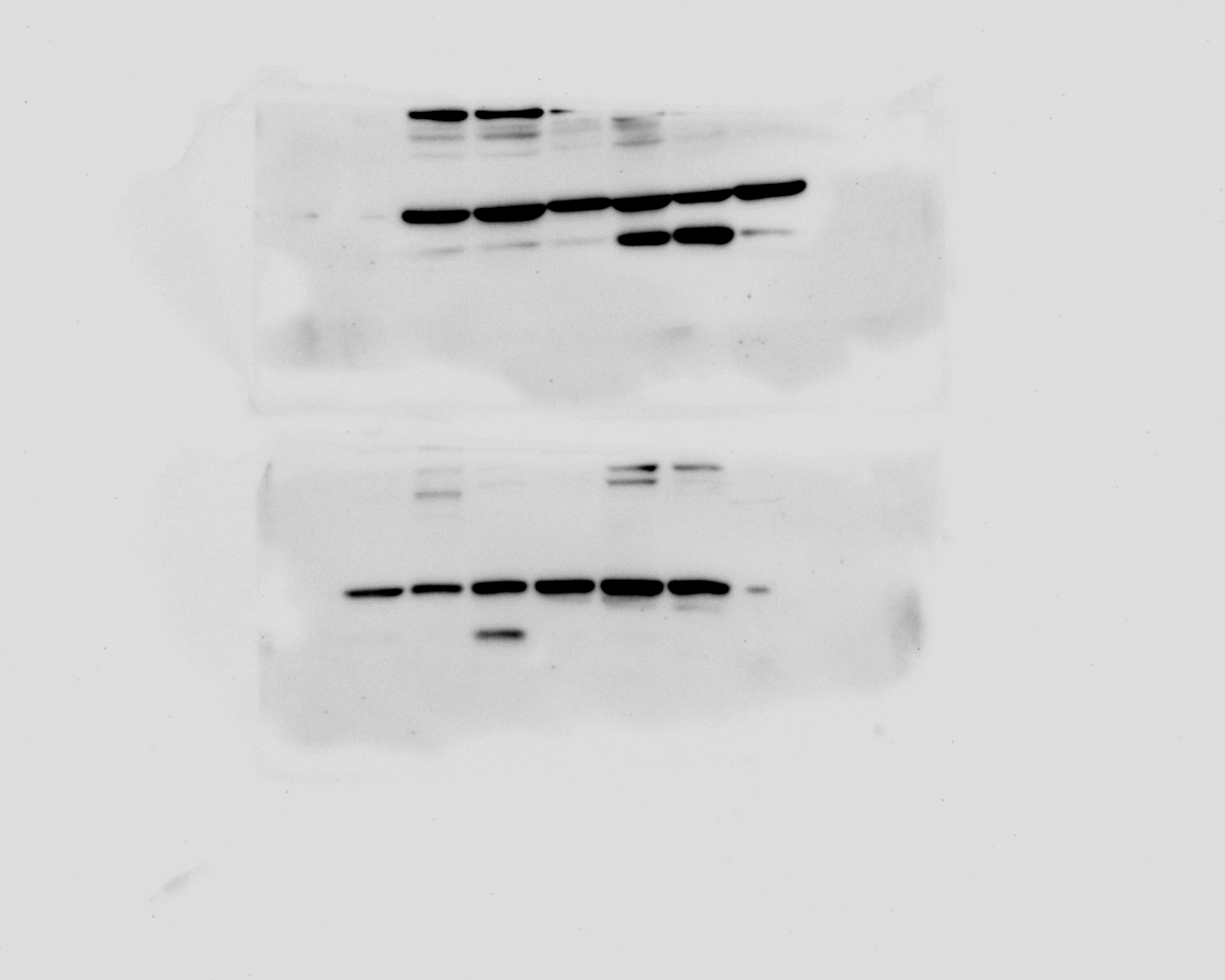


45 kDa

45 kDa

Neo MUC1 Neo MUC1 Neo MUC1

C57MG MOVCAR MC38

Beta-actin

Beta-actin

HPAFII CFPAC BxPc3. MiaPaca2.

Neo MUC1 Neo MUC1

Supplementary Figure 3B. WB on human cell lines.


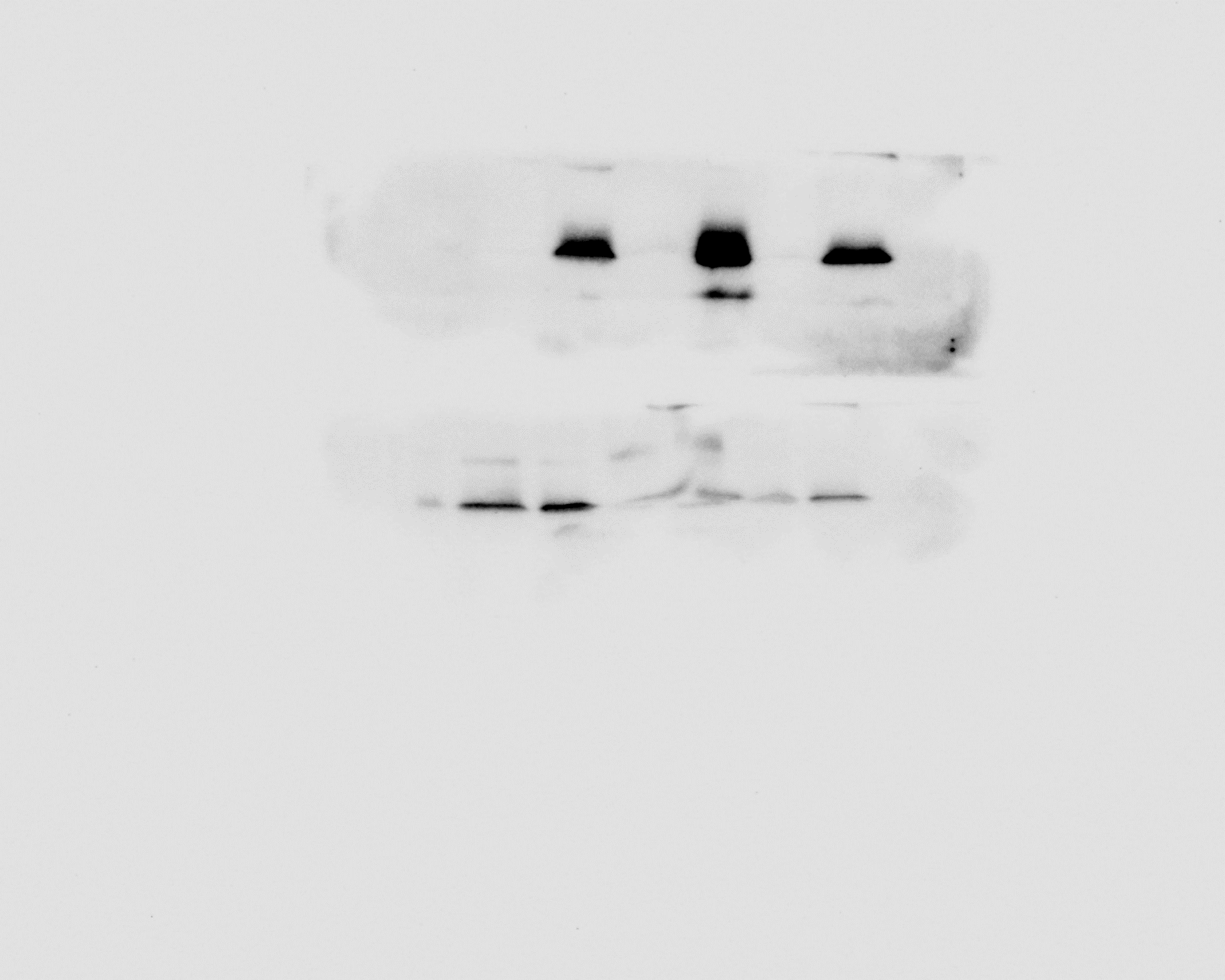


25 kDa

MUC1

Neo MUC1 Neo MUC1 Neo MUC1

C57MG MOVCAR MC38

Supplementary Figure 3B. WB on human cell lines.


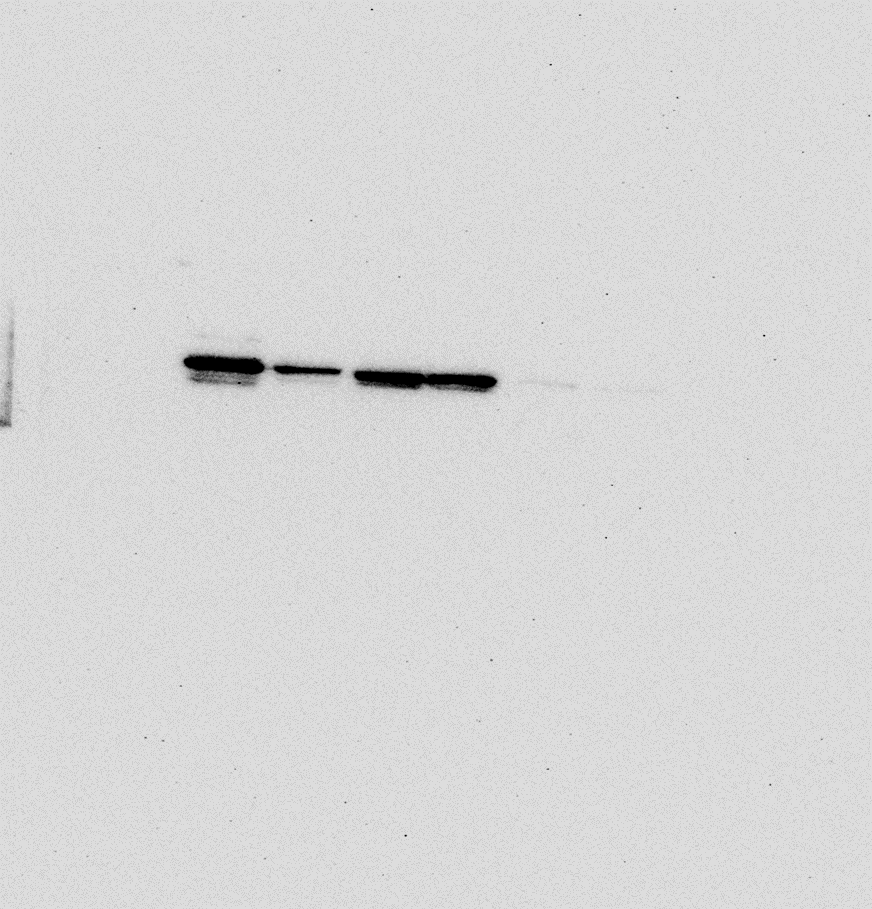


80 kDa

Phospho-STAT3-Y705

UT Nap UT Nap

CFPAC MiaPaca2

Figure 4. Western blot on high MUC1 and low MUC1 cell line.


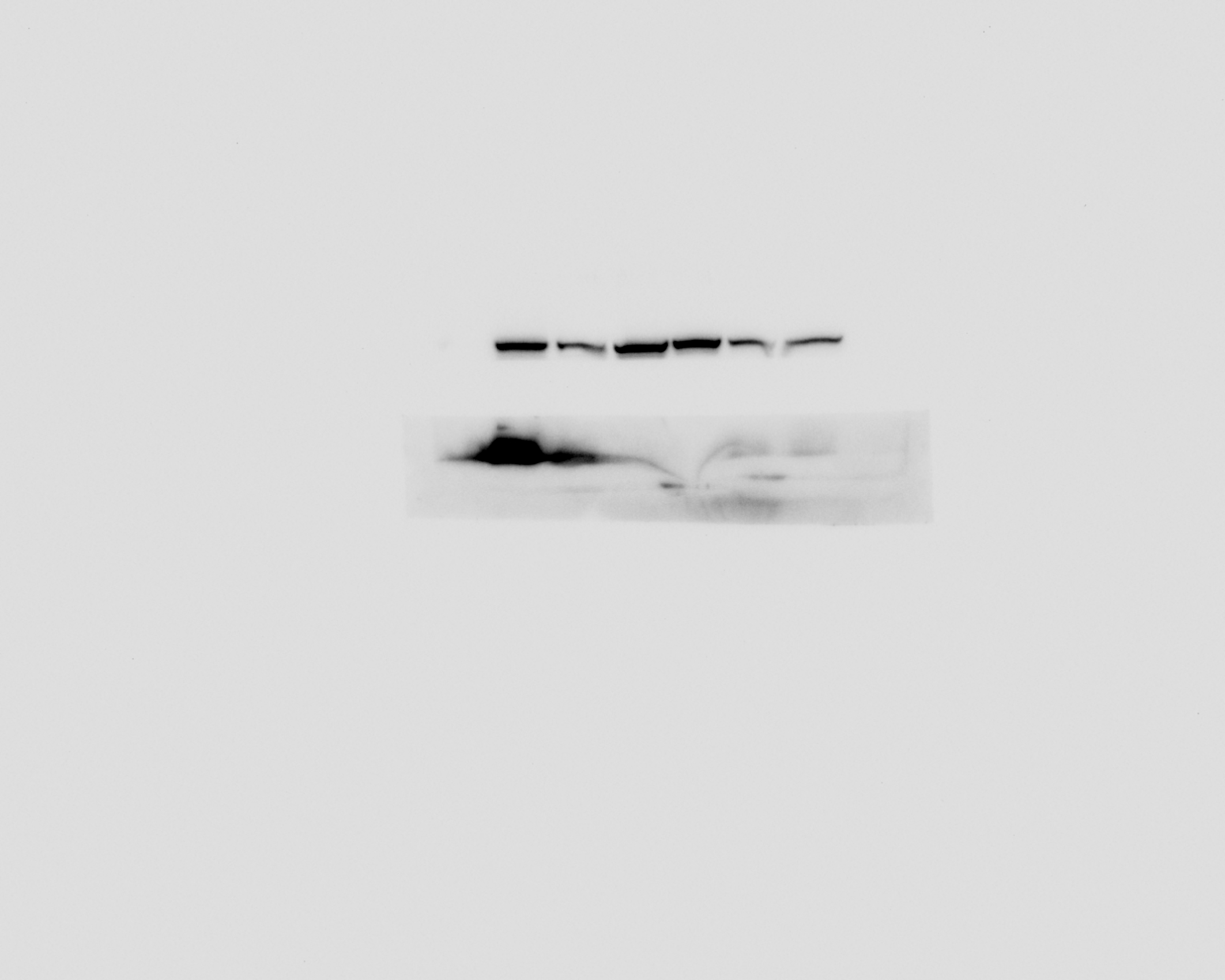


80 kDa

UT Nap UT Nap

CFPAC MiaPaca2

STAT3

Figure 4. Western blot on high MUC1 and low MUC1 cell line.


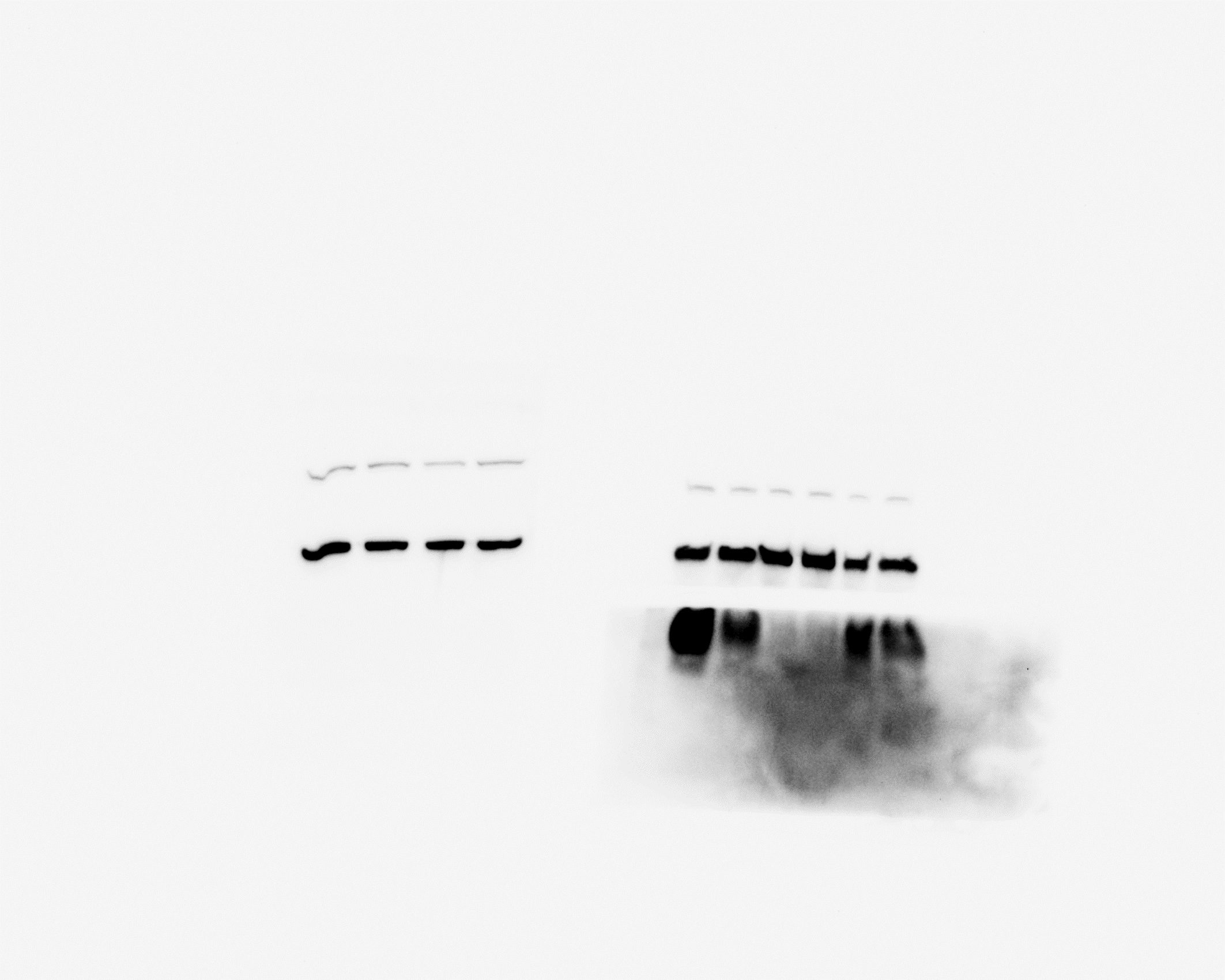


25 kDa

45 kDa

UT Nap UT Nap

CFPAC MiaPaca2

b-actin

MUC1-CT

Figure 4. Western blot on high MUC1 and low MUC1 cell line.
